# Enhanced CO_2_ Uptake in Cobalt-Based MOFs via Textural Optimization and Water-Induced Flexibility

**DOI:** 10.1021/acsami.6c04958

**Published:** 2026-06-10

**Authors:** Mariangela Oggianu, Chiara Busonera, Fabio Manna, Valentina Mameli, Francesca Perra, Virginia Guiotto, Valentina Crocellà, Luciano Atzori, Norberto Masciocchi, Elisabetta Rombi, Carla Cannas, Maria Laura Mercuri

**Affiliations:** † Department of Chemical and Geological Sciences, 3111 University of Cagliari, S.S.554 bivio per Sestu, Monserrato, Cagliari 09042, Italy; ‡ Consorzio Interuniversitario Nazionale per la Scienza e Tecnologia dei Materiali (INSTM), Via Giuseppe Giusti 9, Firenze, Florence 50121, Italy; § Department of Chemistry, NIS and INSTM Reference Centers, 9314 Università di Torino, Turin 10135, Italy; ∥ Dipartimento di Scienza e Alta Tecnologia and To.Sca.Lab, Università degli Studi dell’Insubria, via Valleggio 11, Como 22100, Italy

**Keywords:** metal, organic frameworks, CO_2_ capture, physisorbents, humid conditions, dynamic CO_2_ capture, static CO_2_ capture

## Abstract

Developing metal–organic frameworks (MOFs) that combine high CO_2_ uptake, ultramicroporosity, and stability under humid conditions remains a challenge for practical carbon capture. Here, a rational and optimized synthetic strategy allows to eliminate Co­(OH)_2_ impurities, unlocking previously inaccessible ultramicroporosity in a robust cobalt-based MOF, constructed from Co­(II) nodes and the multitopic 3,6 *N*-ditriazolyl-2,5-dihydroxy-1,4-benzoquinone (trz_2_An) linker. This protocol increases the accessible surface area of 50% while preserving a narrow pore size distribution centered at 3.6 Å, critical for CO_2_:N_2_ selectivity. Static CO_2_ adsorption measurements reveal uptake values of 5 and 4 mmol g^–1^ at 0 and 30 °C, respectively. Dynamic breakthrough experiments with 5–10% CO_2_:N_2_ mixtures demonstrate excellent separation performance and stability over 15 cycles, with mild regeneration in N_2_ at room temperature. Notably, the optimized material exhibits a 24% and 46% increase in CO_2_ uptake at 10 and 25 °C, respectively, compared to the nonoptimized analogue. In addition, and notably in contrast to most CO_2_ adsorbents, CO_2_ uptake further increases under humid conditions, reaching a 65% enhancement, highlighting a beneficial role of water in the adsorption process. This increase is reasonably attributed to transient pore opening due to guest-induced linker flexibility, allowing ultramicropore accessibility without compromising structural integrity. These findings demonstrate how targeted synthetic control can activate latent porosity in rigid ultramicroporous MOFs, offering a viable pathway toward CO_2_ capture under realistic operating conditions.

## Graphical abstract

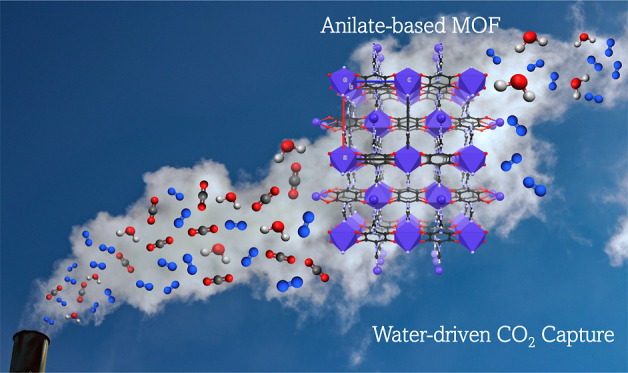

## 1 Introduction

The increasing concentration of atmospheric carbon dioxide (CO_2_) remains one of the most pressing challenges in addressing global climate change. Among the strategies developed to mitigate this issue, carbon capture and storage (CCS) has emerged as a critical approach for reducing CO_2_ emissions from industrial sources.^1–4^ In this context, the design and development of advanced materials with high CO_2_ adsorption capacity and selectivity are of paramount importance to improve the efficiency of CCS technologies. Among these materials, metal–organic frameworks (MOFs),^5–21^ have garnered significant attention due to their high surface area, tunable pore structures, and potential for chemical functionalization. Their customizable structures allow for the integration of functionalities which enhance CO_2_-philic interactions, such as open metal sites or amine grafting. Furthermore, MOFs often rely on physisorption mechanisms, which can offer faster adsorption kinetics and improved regeneration performance, making them attractive for cyclic adsorption-desorption processes.

Understanding their relative performances in terms of adsorption capacity, selectivity, stability, and regeneration under operational parameters miming the real conditions is crucial for designing innovative materials for CO_2_ capture technology. Due to their versatility given by their tunable structures, MOFs offer greater long-term potential, especially with ongoing advancements in stability and scalability. Many MOFs demonstrate stability over hundreds to thousands of CO_2_ adsorption-desorption cycles depending on the specific frameworks and environmental conditions. Advanced MOFs with robust metal-linker bonds and hydrophobic properties have shown exceptional stability, maintaining performance over long operational periods even under humid conditions.^13^

In MOFs, cyclic stability is a multifactorial property that depends not only on the CO_2_ adsorption mechanism (i.e., physisorption or chemisorption), but also on several additional factors, including hydrolytic stability, thermal robustness under regeneration conditions, and mechanical integrity of the framework. In this context, structural features such as open metal sites, ultramicroporosity, and flexible frameworks play a key role in determining both adsorption behavior and long-term stability.^60^ Representative benchmarks for CO_2_ capture, including HKUST-1, SIFSIX-3-M (M = Fe, Co, Ni, Cu, Zn) and ZIF-8, predominantly operate as physisorbents.^22^

It is important to note that, while physisorbents generally exhibit more reversible CO_2_ uptake and therefore favorable cyclic performance under mild regeneration conditions, their stability under real operating environments (e.g., humid or thermally demanding conditions) can vary significantly depending on framework robustness and resistance to hydrolysis. Indeed, even classical physisorbents such as HKUST-1 may show limited cyclic stability under humid conditions.

Generally, MOFs acting as physisorbents showed very high stability over many CO_2_ adsorption–desorption cycles compared to that of open metal sites MOFs and amine-functionalized MOFs, which are also capable of CO_2_ chemisorption^23^ by converting CO_2_ into carbamic acid, carbamate, or bicarbonate species. Most importantly, this physisorption process requires relatively mild regeneration conditions, such as low temperatures and inert gases, due to their reversible CO_2_ sorption properties.^24–26^

The selectivity of MOFs for CO_2_ in physisorption-based processes stems from a combination of kinetic constraints and thermodynamically driven host–guest interactions rather than from molecular sieving behavior and specific intermolecular interactions between the adsorbent framework and gaseous adsorbates. Unlike ideal molecular sieves, which operate purely through rigid steric exclusion based on kinetic diameter, MOFs do not rely exclusively on this mechanism.

Carbon dioxide, owing to its relatively small kinetic diameter and pronounced quadrupole moment, exhibits preferential interactions with polar or electrostatically charged pore surfaces when compared to nonpolar gases such as N_2_ or CH_4_. Notably, microporous MOFs, by virtue of their highly uniform and tunable pore dimensions, facilitate enhanced steric confinement and promote the selective adsorption of CO_2_. This size- and interaction-based exclusion of larger or less polarizable molecules results in markedly high CO_2_:N_2_ and CO_2_:CH_4_ selectivities.^27,28^

Moreover, CO_2_ streams often contain water vapor, meaning that high CO_2_ capacities alone are insufficient for practical applications. However, MOFs sensitivity to moisture remains a significant challenge. In fact, under humid conditions, H_2_O molecules tend to bind more strongly to adsorption sites, competing with CO_2_ and substantially suppressing its uptake. Therefore, the inherently hydrophilic nature of many MOFs further favors water adsorption, resulting in a marked decrease in CO_2_ uptake. This limitation is particularly evident in several high-performing materials, including the MOF-74 family,^29,30^ UTSA-16, and SIFSIX-Mg-HKUST-1.^31,32^ The presence of water may also have a second detrimental effect in terms of MOFs stability, since the metal–ligand coordination bonds can undergo hydrolysis, leading to structural degradation and loss of porosity. Consequently, the majority of studies focus on MOF performance under idealized dry conditions, whereas comprehensive assessments under dynamic adsorption conditions with water vapor remain scarce. This represents a critical knowledge gap, since real-world carbon capture applications inevitably involve humid gas streams, making the evaluation of MOF stability and adsorption performance under such conditions essential for practical implementation.

Recently, a thermally and chemically robust and easily regenerable ultramicroporous 3D physisorbent MOF has been developed by combining Co^II^ metal nodes with the 3,6 *N*-ditriazolyl-2,5-dihydroxy-1,4-benzoquinone, multitopic linker, i.e., trz_2_An anilate.^33^ This linker bears two triazole pendant arms at the 3,6 positions of the anilato moiety and it revealed to be a promising candidate for constructing 3D MOFs, due to the coordinative properties of the N4 atoms of the triazole group (vide infra, Scheme S1). Importantly, the incorporation of multiple polar functional groups within the framework strengthens specific host–guest interactions with CO_2_, thereby enhancing adsorption capacity while simultaneously improving structural stability under humid conditions,^32^ an essential prerequisite for practical carbon capture applications. In addition, the rational design of MOFs with a reduced pore size below 7 Å,^33^ corresponding to the ultramicroporous regime, represents an effective crystal-engineering strategy for achieving high CO_2_ selectivity. Indeed, the material exhibits pronounced molecular sieving behavior, efficiently excluding larger gases such as N_2_ and CH_4_ and delivering outstanding CO_2_ selectivity values (>1000) over a range of temperatures and gas concentrations. Notably, full regeneration can be achieved under remarkably mild conditions without compromising adsorption performance. The synergistic combination of thermal and chemical robustness, high CO_2_ uptake, high selectivity, and facile regenerability highlights the potential of this MOF as a highly efficient molecular sieve for advanced CO_2_ separation.

The aim of this study is to significantly enhance the CO_2_ physisorption performance of this previously reported CoMOF, thanks to the improved synthetic protocol. Specifically, this study aims to (*i*) optimize the synthetic protocol to maximize accessible porosity, (*ii*) correlate structural features and adsorption energetics with static CO_2_ uptake, (*iii*) evaluate CO_2_ capture performance under static and dynamic breakthrough conditions and repeated adsorption–desorption cycles, and (iv) evaluate the effect of humidity on CO_2_ adsorption behavior. By integrating static, dynamic, and humid-condition measurements, this work provides insight into the structure-performance relationships governing efficient and easily regenerable MOF-based physisorbents for carbon capture applications.

## 2 Experimental Section

### 2.1 Chemicals

Trz_2_An (3,6 *N*-ditriazolyl-2,5-dihydroxy-1,4-benzoquinone linker) was prepared according to the literature.^33^ Reagents of analytical grade were purchased from Zentek (TCI) and Sigma-Aldrich and used without further purification.

### 2.2 Synthesis of CoMOF_new_

[Co­(trz_2_An)]*n*·3H_2_O has been synthesized optimizing the synthetic procedure reported in literature for CoMOF_old._ ^33^ CoCl_2_·6H_2_O (23.8 mg, 0.1 mmol) has been slowly added to a mixture of trz_2_An ligand (27.4 mg, 0.1 mmol), NaOH (6 mg, 0.2 mmol) and water 10 mL, and heated in 20 mL autoclave, via a hydrothermal reaction, at 130 °C for 48 h. The dark brown rectangular crystals, suitable for single X-ray diffraction study, were washed three times by using an acid aqueous solution (pH = 5) in order to solubilize and remove the Co­(OH)_2_, obtained during the reaction (Scheme S1). The scale-up of the reaction mixture of 10 times for CoMOF_new_ has been successfully performed in a stainless-steel autoclave of 210 mL volume (Figure S1). Elemental analysis of C_10_H_10_N_6_O_7_Co (385.16), theoretical: C, 31.18; H, 2.62; N, 21.82, experimental: C, 31.12; H, 2.45; N, 22.00.

### 2.3 WA_PXRD

Wide Angle Powder X-ray Diffraction patterns of CoMOF_new_ were collected by using a θ–θ Bragg–Brentano geometry Seifert X 3000 diffractometer equipped with a Cu Kα source (λ = 1.5418 Å), a graphite monochromator on the diffracted beam, and a scintillation counter. Step size 0.05°, acquisition time 2 s step^–1^.

### 2.4 Pore Analysis on CoMOF

The topological analysis was performed with topcryst.com and a standard representation algorithm suitable for coordination compounds and valence-bonded MOFs was selected to simplify the structure. The result was represented by ToposPro. By removing the solvent crystallization molecules, the size and shape of the voids were calculated and represented using the contact surface method by means of the software Mercury (Probe Radius = 1.20 Å or 1.00 Å and Grid Spacing = 0.3 Å). The Pore Analysis was performed by the same software using the following default parameters (Temperature = 298 K; He probe σ = 2.58 Å; N_2_ probe σ = 3.314 Å; He probe ε = 10.22 K; cutoff distance 12.8 Å; cubelet size 0.2 Å; number of samples per atom = 500).

### 2.5 Thermogravimetric Analysis (TGA)

TGA curves were obtained using a PerkinElmer STA 6000. The temperature range investigated was 25–800/850 °C, with a heating rate of 10 °C min^–1^. The measurements were conducted with a flow rate of 40 mL min^–1^ of nitrogen.

Ar-physisorption analysis on CoMOF_new_ was performed at −186 °C using a Micromeritics ASAP2020 sorption analyzer. The cooling bath was prepared using liquid argon. Before the analysis, the sample (about 50 mg) was activated by heating the powder overnight at 80 °C (heating ramp: 3 °C min^–1^) under dynamic vacuum.

CO_2_, N_2_, and H_2_O physisorption analyses on CoMOF_new_ and CoMOF_old_ were carried out using a Micromeritics ASAP 2020 and 3Flex sorption analyzers. To keep isothermal conditions for each analysis, the sample was inserted into a homemade patented glass coating cell in which a coolant or heating fluid, connected to a thermostatic bath (Julabo F25), can recirculate. About 50 mg of sample was activated by heating overnight at 80 °C (heating ramp: 3 °C min^–1^) under dynamic vacuum. Apparent CO_2_-derived BET specific surface area was evaluated following the Rouquerol consistency criteria in the range 1.6 × 10^–2^–2.8 × 10^–2^ *p*/*p* ^0^. Pore size distribution (PSD) and cumulative pore volume (CPV) were calculated through NL-DFT by employing the “CO_2_@273-Carbon” model for a slit pore geometry. For IAST analysis, the N_2_ and CO_2_ isotherms at 30 °C were fitted with the Freundlich–Langmuir equation. The IAST calculation was performed using the software IAST++.

In situ Fourier transform infrared (FT-IR) spectroscopy measurements were performed within the 4000–500 cm^–1^ spectral range using a Bruker Vertex 70 spectrophotometer equipped with a MCT (mercury cadmium tellurium) cryogenic detector. The resolution of the reported spectra is 2.0 cm^–1^ and an average of 32 scans was used to enhance the signal-to-noise ratio. Before the analysis, the sample, in form of self-supported pellet mechanically protected by a gold envelope, was inserted in a homemade quartz cell with KBr windows. To fully activate the material, the pellet was treated at 80 °C (heating ramp: 3 °C min^–1^) overnight under dynamic vacuum by using a conventional high-vacuum glass line, equipped with mechanical and turbo molecular pumps (residual pressure *p* < 10^–4^ mbar).

### 2.6 Microcalorimetry Measurements

Adsorption microcalorimetry measurements were carried out with a Tian Calvet heat flow calorimeter (Setaram, Caluire, France), equipped with a volumetric vacuum line. The samples (ca. 0.1–0.2 g, 20–40 mesh), were pretreated at 60 °C for 12 h under vacuum (ca. 2 × 10^–5^ mbar). Adsorption was carried out by admitting successive doses of CO_2_ at 30 °C. The heat evolved by each dose (Δ*Q*_int,i_) was measured and the corresponding amount adsorbed (Δ*n*_a,i_) was obtained by the pressure drop in the known volume of the apparatus. The equilibrium pressure was measured by means of two differential pressure gauges, operating in the range 0–13.33 and 13.33–1333 mbar, respectively. The CO_2_ amount adsorbed up to the ith dose, ΣΔ*n*_a,i_ (=na), and the corresponding heat evolved, ΣΔ*Q*_int,i_ (=*Q*_int_), were calculated and the volumetric (*n*_a_ vs *P*) and calorimetric (Q_int_ vs P) isotherms were obtained. By plotting the data in terms of *Q*_int_ vs *n*_a_, the differential heat of adsorption, *Q*_diff_, which represents the differential molar enthalpy of adsorption, was determined through the following equation

1 $${\left(Q_{diff}=\frac{{\delta Q}_{\mathrm{int}}}{{\delta n}_{a}}\right)}_{T,m}$$

where *m* is the mass of solid.

A plot of the site energy distribution function vs. *Q*_diff_ can also be drawn. This distribution function is defined as the negative inverse of the first derivative of the differential heat with respect to the amount adsorbed, that is, -d*n*_a_/dQ_diff_. Peaks in this plot correspond to steps in the Q_diff_ vs *n*_a_ curve. The area under each peak is proportional to the amount adsorbed on the family of sites whose adsorption strength corresponds to the abscissa at the peak maximum.

Study of CO_2_ capture performance of CoMOF_new_ Dynamic CO_2_ adsorption measurements were performed using a modified TPD/R/O setup, as shown in Scheme S2.

First, 0.250 g CoMOF_new_ in form of crystalline powder as prepared without any further treatment, were packed between two pieces of quartz wool inside a quartz reactor. Before carrying out the adsorption test, the sample was pretreated at 120 °C for 1 h (heating ramp 2 °C min^–1^) in flowing N_2_ (50 mL min^–1^) to remove moisture and any preadsorbed gases. After pretreatment, the sample was cooled down to the selected adsorption temperature (10/25/30 °C) and exposed to a mixture of CO_2_ (5, 10, 50% vol) in N_2_ (total flow rate of 15 mL min^–1^) for a maximum of 1.5 h. Helium was used as an inert tracer gas (1 mL min^–1^ of He included in the total feed flow of 15 mL min^–1^) to determine the system dead time, which was subsequently subtracted from the breakthrough measurements under each operating condition. Once the adsorption was completed, the gas was switched to pure N_2_ for 1.5 h to regenerate the sorbent. Finally, to evaluate the sorbent regenerability, an additional adsorption–desorption cycle was performed under the same conditions as the initial test (30 °C, 10% CO_2_). The material exhibits full regenerability under mild conditions for 15 consecutive cycles in different conditions, simply by flushing nitrogen at the temperature adopted for the tests. The outlet CO_2_ concentration during the adsorption–desorption steps was continuously monitored by a mass spectrometer (Thermo Scientific ProLab). The tests were performed at least in duplicate (two adsorption–desorption cycles) for each percentage of CO_2_ at each temperature.

To assess the reproducibility in terms of performance, a second batch (batch 2) of CoMOF_new_ was pretreated following the same protocol adopted for the first batch (120 °C for 1h in flowing N_2_ (50 mL min^–1^) and subsequently tested using a mixture of CO_2_ 10% vol. in N_2_ (total flow rate of 15 N mL min^–1^) at 30 °C. After confirming consistent performance for this sample, adsorption measurements were conducted under humid conditions. The experiments were performed at 30 °C using the same gas mixture (10% vol. CO_2_ in N_2_, total flow rate of 15 N mL min ^–1^) at a relative humidity (RH) of 33%, corresponding to the maximum achievable RH under the adopted operating conditions (gas flow rate, temperature, and instrumental setup). Once the adsorption was completed, the sorbent was regenerated in pure N_2_ (15 N mL min^–1^) at 120 °C (heating ramp rate 2 °C min^–1^) for 1 h. Finally, to evaluate the sorbent regenerability after the exposure to a humid gas mixture, an additional adsorption–desorption cycle was performed using a dry CO_2_:N_2_ mixture (10% vol. CO_2_) at 30 °C. The outlet CO_2_ concentration during the adsorption–desorption steps was continuously monitored by a mass spectrometer (Thermo Scientific ProLab). All tests were performed in duplicate (two adsorption–desorption cycles).

## 3 Results and Discussion

### 3.1 CoMOF_new_: Postsynthetic Purification Procedure

Postsynthetic purification procedure of the previously reported CoMOF synthetic protocol,^33^ as described in the Scheme S1 and giving raise to the sample hereafter named as **CoMOF**_**new**_ (Figure S1), was driven by insights from in situ FT-IR spectroscopy following an activation procedure aimed at removing residual solvent species and physisorbed water from the pore channels. The FT-IR spectrum of the as-synthesized CoMOF from the old procedure, named CoMOF_old_ (Figure S2, green spectrum), revealed the presence of water within the channels. This water was partially removed by degassing at room temperature (Figure S2, pale orange spectra) and completely eliminated after heating the sample at 80 °C for 12 h (orange spectrum, Figure S2, also reported in Figure 1a), as evidenced by the disappearance of the broad band in the 3700–3000 cm^–1^ region, characteristic of OH groups. This observation was further supported by a 10% weight loss estimated by thermogravimetric analysis (Figure 1b). The FT-IR spectrum of CoMOF_old_ also showed a sharp signal centered at 3632 cm^–1^ (Figure 1a), which can be attributed to the stretching vibrations ν­(O–H) of Co­(OH)_2_, an impurity that was not detectable in FT-IR spectra acquired at room temperature (Figure S2). To improve the purity of CoMOF, the CoMOF_old_ synthetic protocol was modified by introducing an additional washing step with a mildly acidic solution (pH = 4–5), differing from the procedure described in the previously published work.^33^ The IR spectrum of the resulting material, hereafter named **CoMOF**_**new**_, showed no signal corresponding to ν­(OH) from Co­(OH)_2_, confirming the effective removal of impurities (Figure 1a, blue spectrum). The PXRD patterns of the materials synthesized by using both the original and the optimized protocols were completely superimposable (Figure 1c), not evidencing the presence of Co­(OH)_2_ in the case of CoMOF_old_, likely due to the formation of Co­(OH)_2_ as a thin surface layer and/or its amorphous nature. CO_2_ physisorption measurements (Figure 1d) revealed a significant enhancement in total CO_2_ uptake of CoMOF_new_ compared to CoMOF_old_, corresponding to an increase in apparent CO_2_-derived surface area from 409 ± 4 m^2^ g^–1^ to 617 ± 4 m^2^ g^–1^ (a 51% improvement), supporting the hypothesis of a partial surface coating that previously hindered CO_2_ access to the ultramicropores. It is important to note that CO_2_-derived surface areas may underestimate the true surface area of the material. Therefore, the reported values are most appropriately used for a direct comparison of pore accessibility before and after the washing procedure, rather than for comparison with surface areas of other MOFs determined using conventional probes such as N_2_ or Ar at cryogenic temperatures, which are unable to access the ultramicropores of CoMOF.

**Figure 1:**
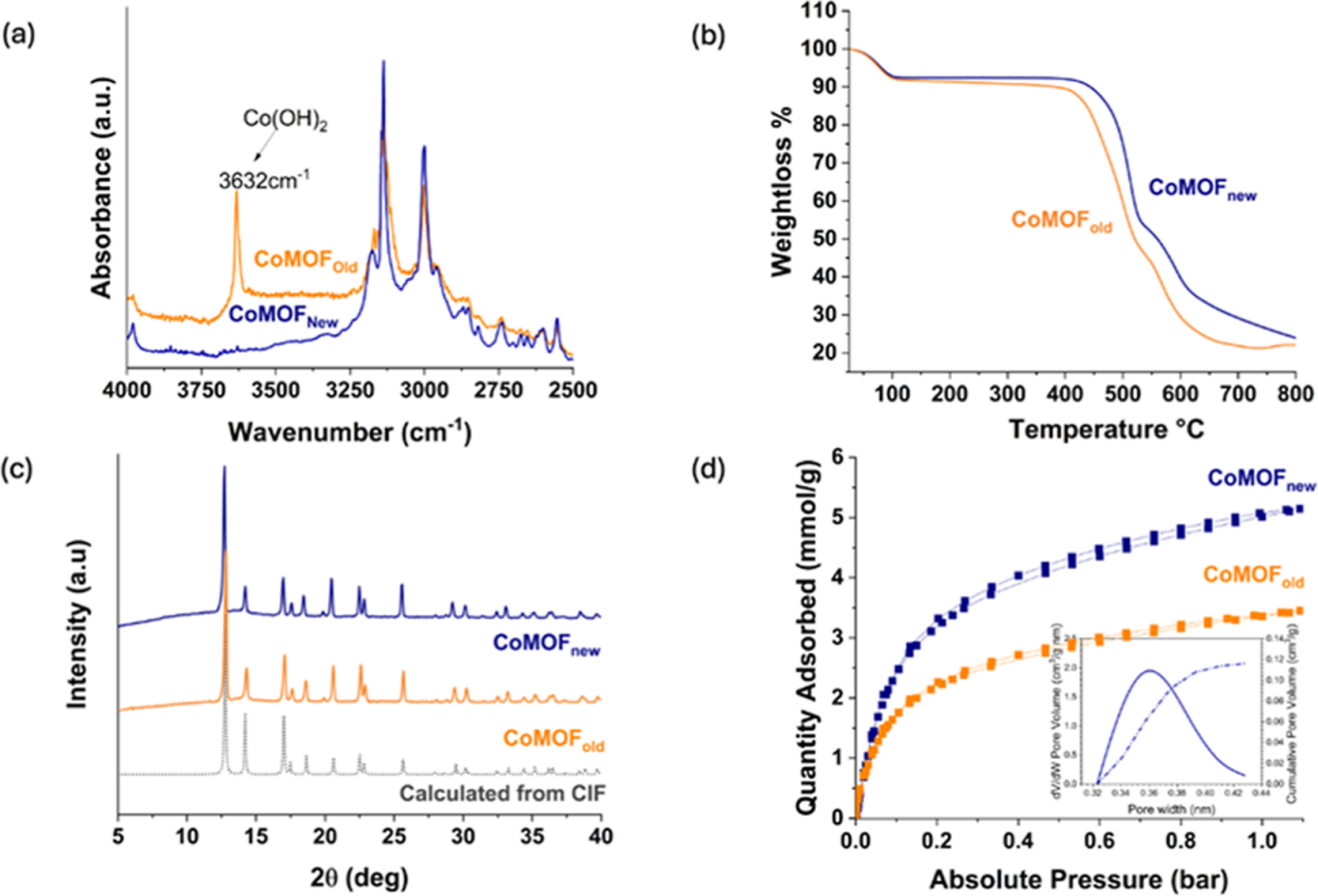
CoMOF before (CoMOF_old_, orange) and after (CoMOF_new_, blue) the synthetic protocol optimization: (a) FT-IR spectra at 80 °C; (b) TGA curves; (c) PXRD patterns compared to that calculated from CIF (dotted black curve); (d) CO_2_ adsorption/desorption isotherms collected at 0 °C and CoMOF_new_ pore size distribution (inset).

The pore size distribution (inset Figure 1d) evidence an average pore size of 3.6 Å with a narrow distribution ranging from 3.2 to 4.2 Å. This enhancement was only observed using CO_2_ as a probe. Even argon (Figure S3), typically considered an ideal probe due to its inertness and spherical shape,^34^ was unable to access the ultramicropores of the CoMOF_new_ framework.

### 3.2 Pore Analysis

Taking into account the CoMOF_old_ structure already reported,^33,35^ this work illustrates the crystal packing and topology (Figure 2), together with the pore analysis (Figure 3), given the crucial role of porosity in the specific application. In this MOF both the metal and the linker act as 4-connected (4-c) nodes arranged at ideal 90° angles, generating a *cds* topology. This topology corresponds to a three-periodic net (vertex symbol 6.6.6.6.6^2^) built from 4-c nodes connected through alternating 90° rotations in three dimensions. Owing to its high symmetry and stability, the *cds* topology, common to many MOFs,^36^ gives rise, in this case, to square channels along the [001] direction, as well as rectangular cavities extending along the [110] and [−110] diagonals, alternating by 90° along the *c* axis. These structural features are shown in Figure 2.

**Figure 2:**
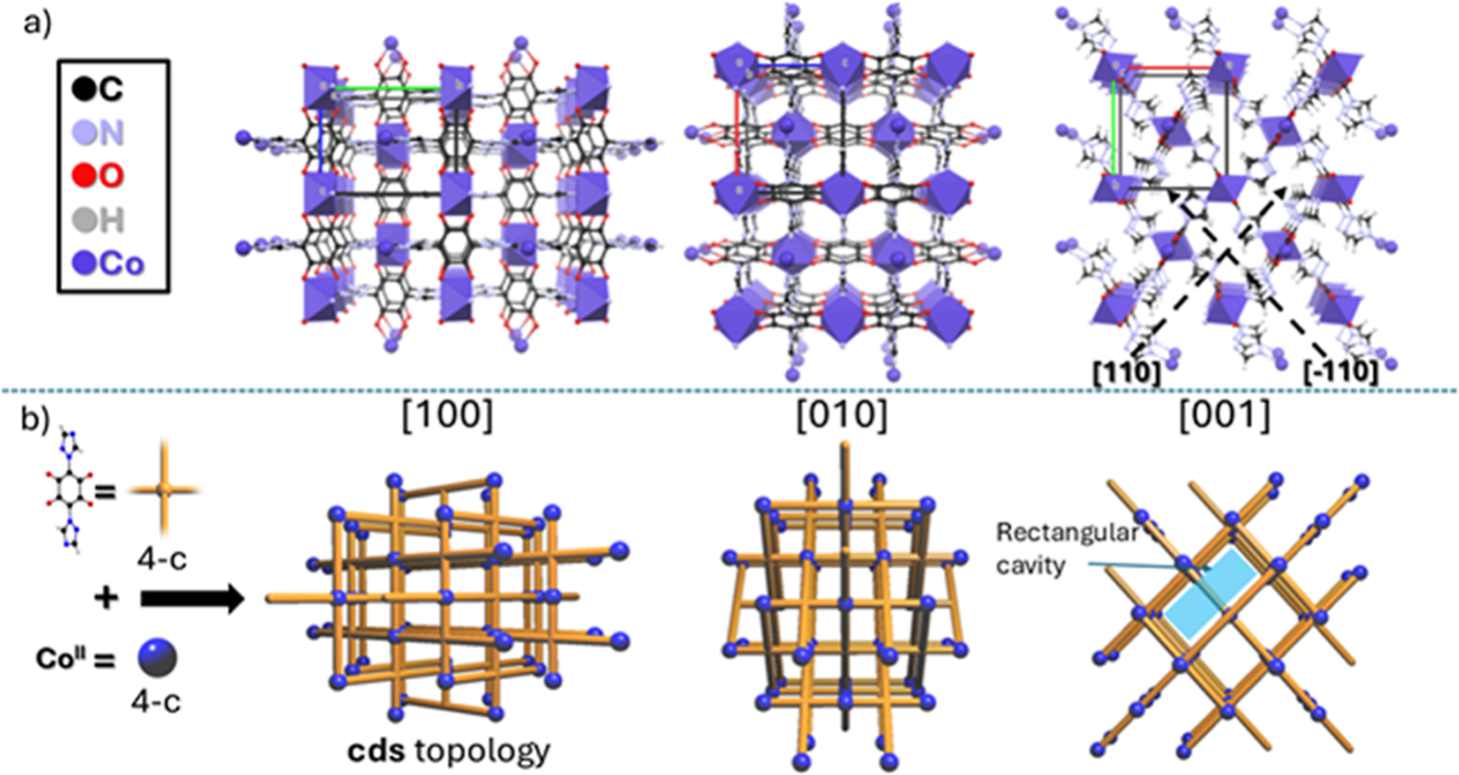
(a) Representation of the crystalline packing of CoMOF; (b) representation of the cds topology obtained using the standard representation algorithm along the main crystallographic directions [100], [010] and [001]. The [110] and [−110] diagonal and rectangular cavity mentioned in the text are highlighted as black arrow and light blue rectangle, respectively.

**Figure 3:**
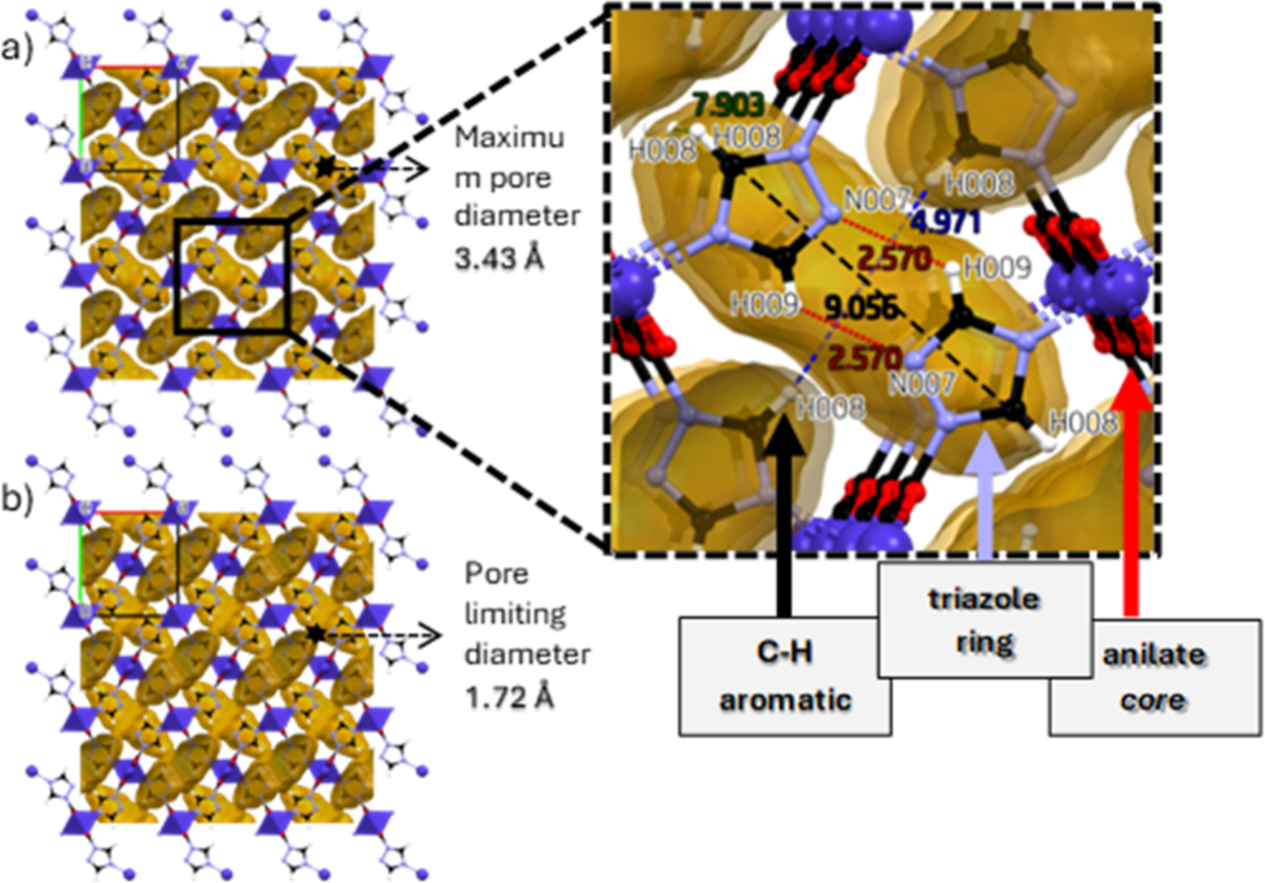
A comparison between the void space calculated with 1.20 Å (a) and 1.00 Å (b) probe radius, where voids descriptor mentioned in the text are highlighted. Zoom of the pore environment where CO_2_ potential interaction sites and distances that describe the cavity dimensions are highlighted (right) (see Supporting Information for a more detailed description). The MOF is represented along the *c* axis.

The void volume was calculated from the crystal structure using the contact surface area method implemented in Mercury,^37^ with a default probe radius of 1.20 Å.^38–41^ The result corresponds to 30% of the unit cell (229.45 Å^3^). The unit cell volume determined by Single Crystal XRD (SCXRD) at −173.15 °C is a reliable approximation of the room-temperature volume, as confirmed by Figure 1c, which compares the diffraction pattern calculated from the.cif file with the powder diffraction pattern collected at room temperature, showing no significant shifts.

The pore analysis indicates the presence of isolated, peanut-like cavities with a volume of 114.7 Å^3^ and a maximum pore diameter of 3.43 Å. Each unit cell contains two such cavities (Figure 3a). The maximum pore diameter (3.43 Å) is in very good agreement with the mean pore size (3.60 Å) determined by CO_2_ physisorption isotherms at 0 °C (Figure 1d, inset). As shown in Figure 3b, each cavity provides multiple sites that can interact favorably with CO_2_, mainly due to the multitopic linker. Specifically, these include groups acting as Lewis acids (H8 atoms, also functioning as H-bond donors), groups capable of π-interactions (triazole rings and the anilate core), and basic groups such as the N atoms of the triazole rings and the O atoms of the anilate core.

From static measurements performed with pure CO_2_ (CO_2_-physisorption isotherms at 0 °C and 1 bar, reported in Figure 1d, and CO_2_ -microcalorimetric measurements at 30 °C and 0.8 bar, shown in Figure 4), the amount of CO_2_ adsorbed ranges from 5.0 mmol g^–1^ (at 0 °C) to 4.2 mmol g^–1^ (at 30 °C), corresponding to approximately 1.39–1.66 CO_2_ molecules per formula unit. These values are in line with geometrical computations (see Supporting Information, ST1: Pore Analysis, Figure S4, Table S1) suggesting the presence of enough space for hosting up to 2 CO_2_ molecules.

**Figure 4:**
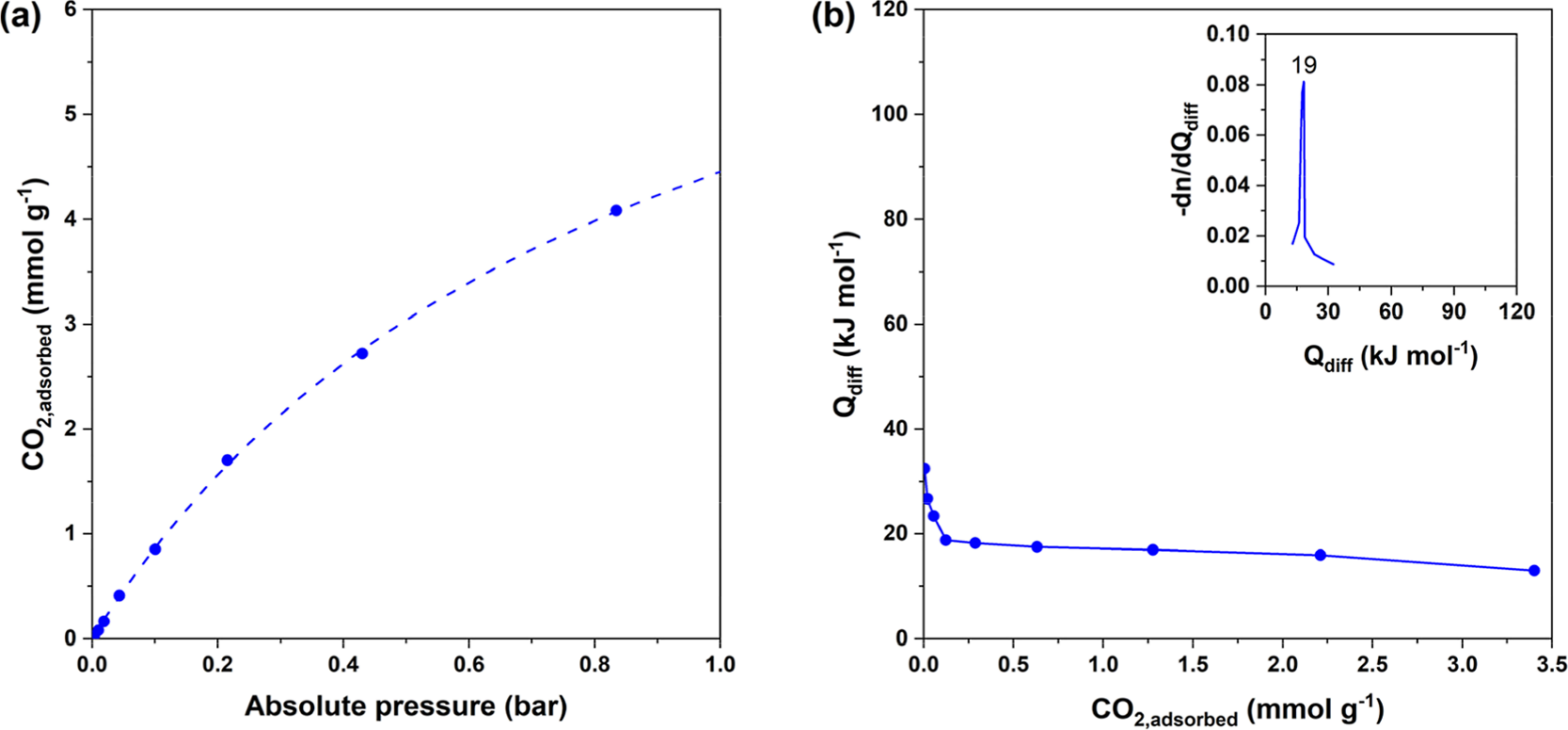
Microcalorimetry measurements of CO_2_ adsorption at 30 °C. Volumetric isotherm (a); Q_diff_ versus CO_2_ uptake (b) and site energy distribution (inset) curves of CoMOF_new_. The dashed line represents the fitting curves of the volumetric isotherms.

As previously described, the framework contains discrete, isolated voids, and analysis with the pore tool identifies a parameter referred to as the pore limiting diameter, which is estimated to be approximately 1.72 Å (Table S1). This value is significantly smaller than the kinetic diameter of CO_2_ (3.3 Å) and other common gases such as N_2_ (3.6 Å) and CH_4_ (3.8 Å).^42,43^ In a rigid structure, such narrow pore apertures would preclude the diffusion of these molecules through the framework. To rationalize the possibility for the CO_2_ molecules to diffuse within the structure, it is necessary to invoke a degree of framework flexibility, as reported also by other authors for CALF-20^13^ and Cu_*4*_I_*4*_[(C_*34*_H_*30*_N_*8*_)^44^ (see Table S1 for comparison). This flexibility allows for transient pore opening and the formation of accessible diffusion pathways. In the present case, pore connectivity is obstructed by 1,2,4-triazole rings that form dimeric hydrogen bonds (Figure 3b). These rings are located along the lateral walls of the cylindrical cavities and at their axial ends, effectively sealing the cavities and preventing communication between adjacent planes in the rigid framework. Upon exposure to a CO_2_:N_2_ gas stream, CO_2_ molecules appear to induce a localized expansion of the limiting pore aperture. This effect could arise from a slight rotation of the triazole rings, which generates transient channels between adjacent cavities with effective diameters exceeding 3.3 Å, sufficient to permit CO_2_ diffusion. In contrast, N_2_ exhibits weaker interactions with the framework and a larger kinetic diameter. These factors, combined with the steric constraints imposed by the narrow pores, prevent N_2_ diffusion. The interplay of framework dynamics, molecular size exclusion, and different host–guest interactions thus accounts for the high CO_2_:N_2_ and CO_2_:N_2_:CH_4_ ^44–47^ selectivity observed in this material. Forcing the probe radius of the software up to 1.0 Å it is possible to represent the described scenario with the interconnections among cavities belonging to different planes and thus resulting in an overall 3D porosity that can justify the diffusion and the observed performances (Figure 3b).

### 3.3 Static CO_2_ Adsorption Properties

The results of the microcalorimetric analyses for CoMOF_new_ are reported in Figure 4. The volumetric isotherm of CoMOF_new_ (Figure 4a) shows that, at 30 °C, the amount of adsorbed CO_2_ continuously increases with pressure over the entire investigated range, the same trend being observed also at 40 °C (Figure S5). The initial value of the differential heat of adsorption (*Q*_diff_, 32 kJ mol^–1^) corresponds to about twice the heat of condensation of CO_2_ at 30 °C (16 kJ mol^–1^),^48^ which can be taken as the threshold value between nonspecific adsorption and chemisorption, generally considered equal to about 2–3 times the heat of condensation.^49^ A similar value was calculated for the near-zero coverage isosteric heat of adsorption of MIL-160 by applying Sips analysis with Clausius–Clapeyron calculations to the isotherm data collected at different temperatures.^50^ Noteworthy, the steep decrease in the differential heat of adsorption below 20 kJ mol^–1^) at low CO_2_ uptakes (*ca*. 0.12 mmol g^–1^) and the presence of a sharp peak centered at 19 kJ mol^–1^ in the site energy distribution plot (Figure 4b, inset) clearly indicate that only weak adsorbent/adsorbate interactions occur.

The volumetric isotherm was fitted by the Freundlich–Langmuir (FL) model (eq 2), as proposed in the literature^14^

2 $$n_{a}=\frac{a_{1}\cdot b_{1}\cdot P^{c}}{1+b_{1}\cdot P^{c}}$$

where *n*_a_ is the amount adsorbed (mmol g^–1^), *P* is the pressure (bar), *a*_1_ is the maximum CO_2_ loading (mmol g^–1^), *b*_1_is the affinity constant (bar ^–c^), and c is the heterogeneity exponent. To compare static (volumetric isotherms) and dynamic (breakthrough curves, later described in the manuscript) adsorption measurements, only the CO_2_ amounts adsorbed with Q_diff_ values beyond 16 kJ mol^–1^, i.e., prior to condensation, were considered in the fitting procedure. Indeed, it can be reasonably assumed that under dynamic testing conditions, where the gas stream is continuously fed, CO_2_ condensation does not occur to any appreciable extent. The values of the fitting parameters are reported in Table S2.

Similar trends in the amount of CO_2_ adsorbed as a function of pressure were observed at atmospheric pressure and temperatures in the range 20–30 °C for MIL-160,^14^ MIL-160­(Al),^51^ mCB-MOF-1,^19^ MOF-74­(Ni) and MOF-74­(Co),^6^ for which CO_2_ uptakes of 4.3, 3.2, 1.4, 4.1, and 3.7 mmol g^–1^ were calculated, respectively. Comparable values of CO_2_ adsorbed amounts were also reported for CALF-20 (4.1 mmol g^–1^),^13^ UTSA-120 (5.0 mmol g^–1^),^52^ TAMOF-1 (3.8 mmol g^–1^),^7^ and MIL-120­(Al)-AP (1.9 mmol g^–1^).^53^ To the best of the present authors’ knowledge, only the Mg-MOF74 sorbent exhibited a remarkably superior performance (8.2 mmol g^–1^),^54^ due to the ability of this material to act both as a molecular sieve and by chemisorption through the strong interaction of CO_2_ with the open Mg^II^ sites.

A comparison of the CO_2_ adsorption performances of the different MOFs in static conditions is reported in Table S3. Overall, the observed value for CoMOF_new_ at 30 °C and atmospheric pressure (4.45 mmol g^–1^) is comparable with those of the most promising MOFs, proving the very good performance of this sample in static conditions.^59^ Furthermore, CO_2_:N_2_ IAST selectivity was calculated considering two gas compositions (5% and 10% CO_2_ in N_2_). At 1 bar both mixtures yield comparable selectivity values of around 75, which allows considering CoMOF a selective adsorbent for CO_2_ under the investigated conditions (Figure S6). The H_2_O isotherm (Figure S7) indicates a total water uptake of 15 mmol g^–1^ at 90% RH. At higher RH values a steep increase is observed due to external water condensation. The uptake value at 33% of RH, used for wet dynamic analysis, corresponds to 7 mmol g^–1^ of H_2_O, namely about two water molecules per Co^II^ node.

### 3.4 Dynamic CO_2_ Adsorption Properties

Dynamic adsorption measurements conducted using a custom-built apparatus enabled the acquisition of breakthrough curves (BTCs, Scheme S2), which simulate the behavior of the sorbents under realistic gas adsorption conditions. Figure S8 shows the breakthrough profiles (see also Supporting Information **ST2**) of the investigated sorbents at three operating temperatures (10 °C, 25 °C, and 30 °C), using three CO_2_:N_2_ mixtures (10:90,5:95 and 50:50).

The dynamic CO_2_ adsorption results of the CoMOF_new_ sample (Figure S9), highlight its very promising performance, with a significant improvement compared to the good adsorption capacity already reported for CoMOF_old_. This enhancement can be ascribed to the postsynthetic purification procedure proposed in this study. In particular, the additional purification step (Scheme S1) leads to a 51% increase in surface area due to the increase in the pore volume, which in turn improves the adsorption capacity with respect to that of CoMOF_old_ by 24% at 10 °C and 46% at 25 °C (Figure S9). As expected, the adsorption performance of the CoMOF_new_ sample improves markedly with decreasing temperature for both gas mixtures (5% and 10% CO_2_:N_2_) (Table 1 and Figure 5a), with the enhancement particularly pronounced at the lower CO_2_ concentration. At 30 °C, the 50% CO_2_:N_2_ mixture exhibits the highest CO_2_ uptake, reaching 2.18 mmol g^–1^ (Table 1).

**Table 1:** CoMOF_new_ Dynamic Adsorption Capacities (mmol g^–1^) with Different CO_2_:N_2_ Concentrations (5:95, 10:90 and 50:50) and Temperature (10 °C, 25 °C, 30 °C)

sorbent	CO_2_ vol %	adsorption temperature (°C)	CO_2_ adsorbed (mmol g^–1^) dry conditions	CO_2_ adsorbed (mmol g^–1^) Humid conditions RH = 33%
CoMOF_new_	5	10	0.86 ± 0.07	--
		25	0.48 ± 0.02	--
		30	0.32 ± 0.01	0.66 ± 0.02
	10	10	1.06 ± 0.05	--
		25	0.87 ± 0.01	--
		30	0.73 ± 0.02 (batch 1) 0.71 ± 0.04 (batch 2)	-- 1.17 ± 0.07 (batch 2)
	50	30	2.18 ± 0.33 (batch 2)	--

**Figure 5:**
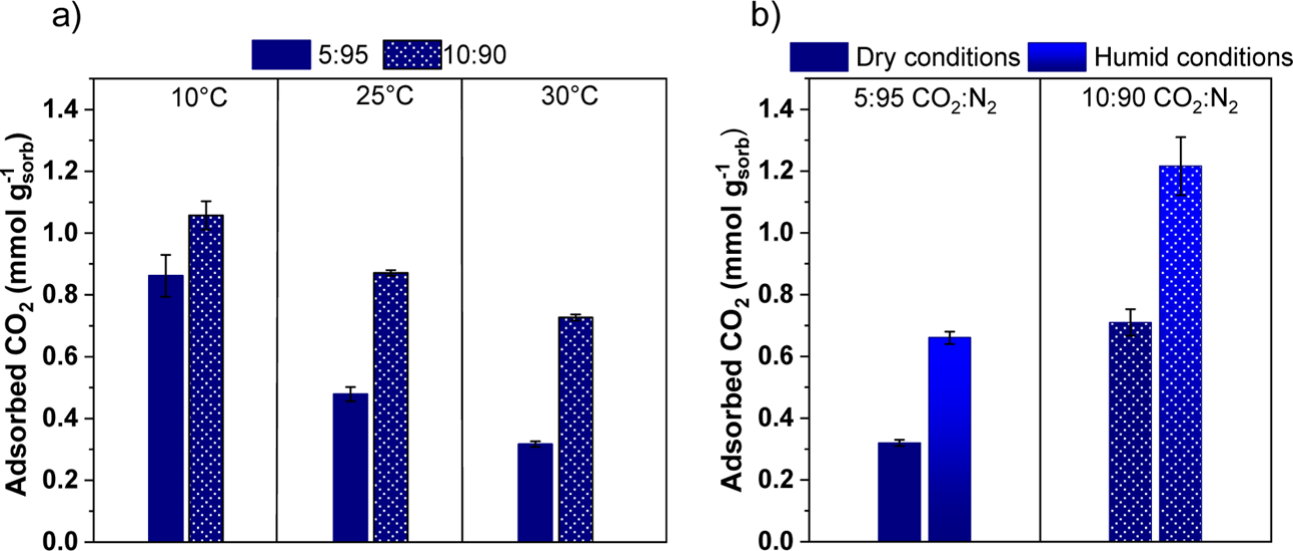
CO_2_ uptake on CoMOF_new_ calculated from breakthrough curves: adsorption measured using different CO_2_:N_2_ gaseous mixtures (5:95 and 10:90) (a) at different temperatures (10 °C, 25 °C, and 30 °C); (b) under dry and humid conditions (RH = 33%).

Notably, CoMOF_new_ exhibits full regenerability under mild conditions for 15 consecutive cycles, simply by flushing nitrogen at the temperature adopted for the tests (Figure S10) for 90 min at a flow rate of 15 mL min^–1^. Postcycling PXRD patterns confirm the retention of the crystalline structure (Figure S11a), and thermal analysis evidence the excellent thermal stability of the system Figure S11b).

Noteworthy, a CO_2_ uptake of 0.73 mmol g^–1^ is obtained at 30 °C for CoMOF_new_ (batch 1) under dynamic conditions using a 10:90 CO_2_:N_2_ inlet gas mixture (Table 1). A second batch of CoMOF_new_ (batch 2) was tested in the same conditions, showing, within experimental error, a value matching that of the initial batch (batch 2, 0.71 mmol g^–1^), confirming the reproducibility of both the synthesis and the CO_2_ capture tests (Table 1). At 30 °C and atmospheric pressure, the dynamic CO_2_ uptakes obtained for CoMOF_new_ using 5:95 (0.32 mmol g^–1^) and 10:90 (0.73 mmol g^–1^) CO_2_:N_2_ inlet gas mixtures are lower than those estimated in static conditions at 0.05 bar (0.46 mmol g^–1^) and 0.1 bar (0.86 mmol g^–1^), i.e. at CO_2_ pressures corresponding to p_CO2_ in dynamic tests. This finding indicates that thermodynamic equilibrium is not reached under dynamic conditions, due to the complex interplay between adsorption kinetics and intraparticle mass-transfer limitations, in agreement with what is generally reported in the literature.

To assess practical applications of CoMOF_new_, further breakthrough experiments were carried out on CoMOF_new_ (batch 2), under humid conditions, with a Relative Humidity (RH) of ∼33%. As reported in Figure 5b and Table 1. Using a 10:90 CO_2_:N_2_ inlet gas at 30 °C,·CoMOF_new_ exhibited a significant increase of CO_2_ uptake performance, from 0.71 mmol g^–1^ (dry) to 1.17 mmol g^–1^ (humid) with a remarkable improvement of 65% (Figure 5b). For the 5:95 CO_2_:N_2_ mixture, under humid conditions the capacity increased from 0.32 to 0.66 mmol g^–1^, corresponding to an improvement of approximately 106% (Figure 5b). These results indicate that the beneficial effect observed in the presence of water is preserved also at low CO_2_ partial pressures, suggesting the positive influence of water molecules on the CO_2_ uptake for this MOF.

The adsorption performance remained stable across multiple humid tests and demonstrated full regenerability over dry-humid-dry cycles (Figure 6).

**Figure 6:**
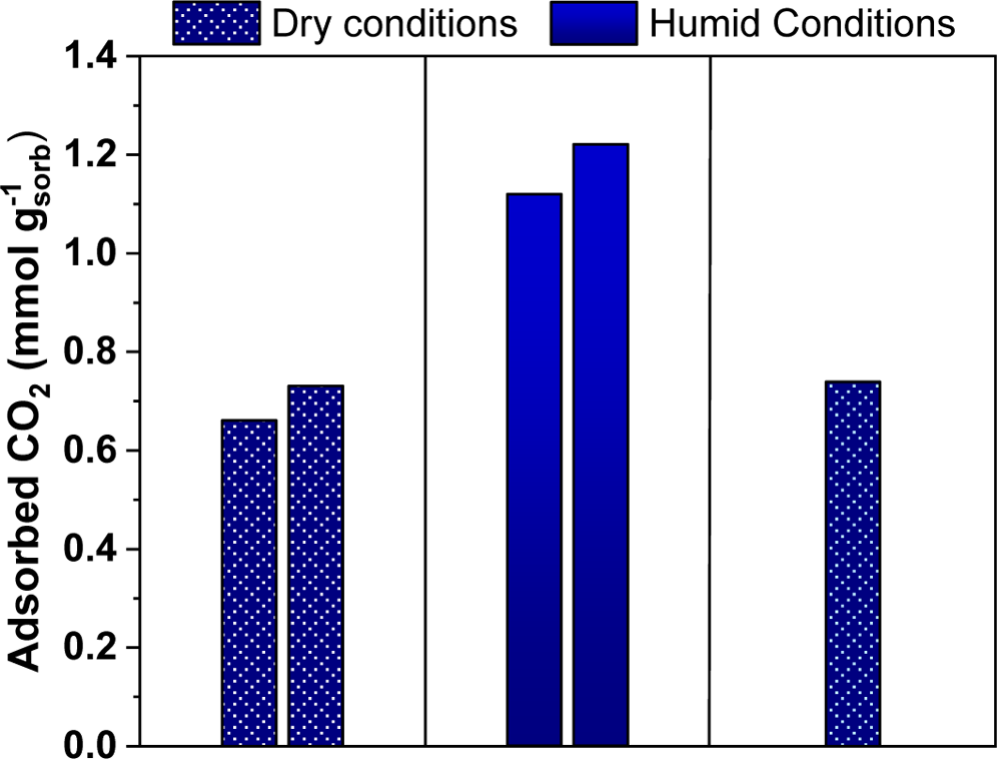
CO_2_ uptake on CoMOF_new_ over dry-humid-dry regenerability cycles using 10:90 CO_2_:N_2_ gaseous mixtures at 30 °C.

We attribute this improvement to a linker flexibility induced by water through cooperative adsorption, which enhances the accessibility of the porous network,^55–57^ which is however not observed in the PXRD sequence supplied in the Supporting Information (Supporting Information ST3, Figures S12–S15 and Table S4). Indeed, the constancy of the raw PXRD data, not significantly modified by water removal or reinsertion, can be (easily) misinterpreted by the absence of linker flexibility. To further corroborate the above statements, we note that the cell volumes, determined by structureless Pawley-type refinements,^58^ change by only 1 Å upon water elimination and reinsertion and confirm the absence of evident structural (ordered) water molecules (a single H_2_O moiety accounts for ca. 25–30 Å^3^).^59^ The very minor (<0.3%) countercorrelated changes of the a and *b* axes (the strain tensor being represented in Figure S15), also prove the significant stiffness of the crystal lattice in static conditions. Altogether, this makes the hydrated material a nice example of channel hydrates, often nonstoichiometric, which have found large applications in the pharmaceutical field.^59^ Therefore, we can conclude that water molecules, where present, behave very much as nitrogen molecules in “empty” MOFs and that, in conjunction with sorption measurements, ligands do possess the sufficient flexibility to enable CO_2_ capture. Accordingly, such (PXRD-derived and apparent) static picture can be changed if dynamics are taken into account, an effect that diffraction methods alone cannot perceive.

A summary of selected literature results on MOFs for CO_2_ adsorption is provided in Table S3. Although a direct comparison with the literature data was attempted, it should be noted that such comparison is not always straightforward. Indeed, dynamic adsorption performance is generally strongly influenced by testing conditions (e.g., temperature, pressure, CO_2_ concentration, and flow rate), as well as by the experimental setup and the nature of the interactions that sorbents can establish with CO_2_ −whether purely chemical, physical, or a combination of both. For this reason, a meaningful comparison between CoMOF_new_ and other MOFs reported in the literature is limited to three samples −MUV-a26, mCB-MOF-1, and CoMOF_old_ ^17,19^ for which the interactions with CO_2_ can be considered exclusively physical and that were tested using the same instrument (ABR, HIDEN Isochema), albeit under different CO_2_:N_2_ gas mixture compositions. Specifically, mCB-MOF1 and CoMOF_old_ tested using a 5:95 CO_2_:N_2_ mixture showed adsorption capacities of 0.09 and 0.33 mmol g^–1^ at 25 °C,^19,33^ respectively (Table S3). Notably, CoMOF_new_ exhibits an improved performance (0.48 mmol g^–1^) under the same experimental conditions (Table S3). A remarkable higher amount of CO_2_ is adsorbed at 25 °C by MUV-a26 (0.81 mmol g^–1^);^17^ however, taking into account the influence of CO_2_ content on the dynamic adsorption behavior, this result can be ascribed to the much higher CO_2_ concentration (50:50 CO_2_:N_2_) in the gaseous stream. MOF-74 family and CALF-20 are considered among the most promising MOFs reported in the literature.

Particularly, Mg-MOF74 was characterized by a very high adsorption capacity at 25 °C (2.32 mmol g^–1^, 15:85 CO_2_:N_2_)^5^ due to strong interactions with open Mg^II^ sites. CALF-20 ^60^ also showed very good performance at *ca*. 25 °C (1.51 mmol g^–1^ at 5:95 CO_2_:N_2_) reasonably due to a strong physisorption as suggested by its isosteric heat of adsorption (*Q*_s_ ≈40 kJ mol^–1^),^61,62^ comparable with that of Mg-MOF74.^5^ Although the dynamic adsorption capacities of Mg-MOF74 and CALF-20 are significantly better than those of CoMOF_new_, the ease of regeneration of CoMOF should also be considered. Indeed, complete regeneration (tested over 15 cycles) of the MOF is achieved through a simple pressure swing process (90 min at a N_2_ flow rate of 15 mL min^–1^ at the temperature of the CO_2_ capture test) without the need of any temperature increase. In contrast, regeneration procedures reported for CALF-20 involve more severe conditions (12 h at 150 °C under He flow). Therefore, despite its lower CO_2_ capacity, the facile regeneration of CoMOF makes it a competitive candidate in terms of practical working capacity compared to other sorbents. Only a few other examples showed comparable behavior (Table S3): CoMOF_old_ (40 cycles),^33^ MUF-16 (12 cycles),^11^ Fe_2_(BDP)_3_ (11 cycles),^63^ and MUV-a26 (10 cycles),^17^ although in different regeneration conditions.

As previously highlighted, and as expected, the adsorption capacity of CoMOF_new_ increases with decreasing temperature and with higher CO_2_ concentrations; however, due to the physisorptive nature of CO_2_ binding, CoMOF_new_ shows greater sensitivity to these changes with respect to chemisorbents. In particular, the amount of adsorbed CO_2_ decreases significantly when the CO_2_ concentration diminishes from 10% to 5%, especially at 30 °C (Figure 5). Hence, CoMOF_new_ demonstrates the most promising adsorption capacity at 10% CO_2_ and offers the advantage of an easy regeneration under mild conditions.

Only a limited number of physisorbent ultramicroporous MOFs, summarized in Table S3, was systematically evaluated under humid conditions. Based on their response to moisture, these materials can be grouped into three categories: (i) negligible variation in CO_2_ uptake; (ii) decreased CO_2_ uptake; and (iii) enhanced CO_2_ uptake.

Some materials, such as mCB-MOF-1 ^19^ and MOF 303-Al,^64^ show almost no change in dynamic CO_2_ adsorption, maintaining capacities of 0.09 mmol g^–1^ (5:95 CO_2_:N_2_) and 0.36 mmol g^–1^ (1:99 CO_2_:N_2_), respectively, even in the presence of moisture. In contrast, several MOFs experience moderate reductions in adsorption under humidity. For instance, for IISERP-MOF2,^16^ tested with a 14:86 CO_2_:N_2_ gas mixture, the uptake decreases from 3.97 mmol g^–1^ to 3.68 mmol g^–1^ (∼8%), while for TAMOF-1,^7^ using a 50:50 CO_2_:CH_4_ mixture, it drops from 2.71 mmol g^–1^ to 2.31 mmol g^–1^ (∼17%). Similar decreases with respect to IISERP-MOF2 are observed for Ni TMDMOF^65^ (15:85 CO_2_:N_2_), whose adsorption capacity declines from 0.88 mmol g^–1^ to 0.83 mmol g^–1^ (∼6%), and for Qc-5-*M*-sql B­(15:85 CO_2_:N_2_),^9^ which shows a reduction from 1.98 mmol g^–1^ to 1.85 mmol g^–1^ (∼7%).

Interestingly, some MOFs exhibit an opposite trend, with water actually enhancing CO_2_ adsorption, as observed for the CoMOF_new_. CALF-20 MOF, for example, shows a humidity-dependent increase: theoretical calculations predict higher CO_2_ uptake at relative humidities up to 20%, although adsorption decreases above this threshold. MgCUK-1,^66^ tested under static conditions, demonstrates a modest increase from 3.0 mmol g^–1^ to 3.3 mmol g^–1^ (∼10%), while CuMOF,^67^ evaluated using breakthrough experiments with a 50:50 CO_2_:N_2_ mixture, shows a slight enhancement from 1.63 mmol g^–1^ to 1.78 mmol g^–1^ (∼9%).

These observations highlight that the impact of water on CO_2_ adsorption is highly MOF-specific, with some frameworks retaining their performance, others experiencing moderate losses, and a few even benefiting from humidity. This complex behavior evidence the importance of systematic, dynamic evaluations under realistic humid conditions, which are essential for understanding the mechanisms governing MOFs stability and adsorption performance and identifying those suitable for practical carbon capture applications.

On the basis of its features and performances, it emerges that the investigated CoMOF can be used more efficiently in carbon capture strategies involving gas mixtures with high CO_2_ concentrations and total pressures even under humid conditions (up 30 RH %). Specifically, CoMOF_new_ could be effectively used in biogas upgrading processes, aimed at removing carbon dioxide from high pressure gas mixtures primarily composed of CH_4_ (40–70 vol %) and CO_2_ (25–45 vol %).^68^ Indeed, the sorbent maintains high adsorption performance even under low CO_2_ partial pressures and its use can be particularly advantageous due to the possibility of performing low energy regeneration cycles. CoMOF could also be investigated as a possible capturing agent in precombustion carbon capture strategies, where fossil fuels are first converted into syngas, which, in turn, is subjected to water gas shift processes to obtain a high pressure (15–30 bar) gas mixture rich in H_2_ (*ca*. 55 vol %) and CO_2_ (*ca*. 40 vol %),^69^ once the selectivity of CO_2_ over H_2_ is demonstrated.

## 4 Conclusion

This study demonstrates that rational synthetic control can significantly modulate the functional performance of ultramicroporous MOFs without modifying their underlying topology. By eliminating residual Co­(OH)_2_ impurities, previously inaccessible porosity was activated, leading to a substantial enhancement in accessible surface area and dynamic CO_2_ capture performance. Besides the quantitative improvement in uptake, the results reveal a more significant conceptual insight: ultramicroporous physisorbent frameworks, often considered structurally rigid, may exhibit adaptive behavior under operating conditions, as discussed in the pore analysis based on the MOF’s structure. Furthermore, the observed humidity-induced enhancement of CO_2_ capture highlights a guest-triggered flexibility mechanism, whereby water molecules stabilize a more open linker conformation, facilitating transient pore accessibility while preserving structural integrity. This adaptive response challenges the conventional view of water solely as a competitive adsorbate in physisorbent MOFs. Under realistic dynamic conditions (5–10% CO_2_ in N_2_), CoMOF_new_ combines high adsorption capacity with complete regenerability under mild conditions, distinguishing it from strongly chemisorptive systems that require energy-intensive regeneration. Although benchmark chemisorptive MOFs exhibit higher absolute CO_2_ capacities, their stronger adsorbate–framework interactions are associated with elevated adsorption enthalpies and consequently higher energetic requirements for regeneration. In contrast, the moderate heat of adsorption and structural robustness of CoMOF_new_ enable efficient CO_2_ capture combined with facile desorption under mild conditions, thereby supporting enhanced cyclic stability and reduced regeneration energy demand. Overall, the combination of selective ultramicroporosity, water tolerance, adaptive pore accessibility, and mild regenerability makes CoMOF_new_ a promising candidate for CO_2_ capture in moderately concentrated and humid gas streams. More broadly, the identification of a water-assisted gating mechanism suggests new opportunities for designing adaptive physisorbent materials tailored for realistic separation environments.

## Supplementary Material



## Notes

Scheme S1. Postsynthetic purification procedure for CoMOF_new_; Figure S1. Calculated and experimental PXRD patterns of CoMOF_new_; Scheme S2. Schematic representation of the homemade apparatus for breakthrough measurements; Figure S2. *In situ* FT-IR spectra of as synthesized CoMOF_old_; Figure S3. Ar adsorption/desorption isotherm of CoMOF_new_; **Text ST1**: Pore Analysis; Figure S4. Volume of a single cavity; CO_2_ volume and parameter for one molecule with cylindrical and ellipsoid shape CO_2_ and volume and parameter for one molecule with ellipsoidal shape; Table S1. Pore parameters calculated for Co­(trz_2_An), CALF-20 & Cu_4_I_4_[(C_34_H_30_N_8_); Scheme S3. (a) Comparison between the adsorption energy diagrams for chemisorption, physisorption, and “magic door” mechanisms, (b) proposed mechanism for CoMOF; Figure S5. CO_2_ adsorption isotherms of CoMOF_new_ and differential molar heat of adsorption at 40 °C; Table S2. Values of the parameters obtained by fitting the volumetric isotherms of CoMOF_new_ with the Freundlich–Langmuir (FL) model; Table S3. State of art for MOFs for CO_2_ capture: Linker, node, textural properties, static and dynamic CO_2_ uptake; Figure S6. CO_2_ and N_2_ adsorption/desorption isotherms on CoMOF_new_ at 30 °C; Figure S7. H_2_O adsorption/desorption isotherms on CoMOF_new_ at 30 °C; Figure S8. Breakthrough curves acquired at different temperatures and CO_2_ inlet concentrations for CoMOF_new_; **Text ST2.** Breakthrough curve Analysis; Figure S9. Comparison of CO_2_ uptake on CoMOF_old_ (orange) and CoMOF_new_ (blue) for the 5:95 CO_2_:N_2_ mixture at 10 and 25 °C; Figure S10. Cyclic stability of CoMOF_new_ evaluated over 15 regeneration cycles; Figure S11. Pattern WA-PXRD and thermogram of CoMOF_new_ before Dynamic Adsorption Test and after 15 adsorption–desorption cycles; **Text ST3.** The effect of water elimination and reinsertion; Figure S12. Raw data for CoMOF_new_ as prepared, dehydrated and rehydrated; Figure S13. Raw XRPD data for CoMOF_new_ as prepared, dehydrated and rehydrated, plotted in the limited range; Figure S14. Pawley-type full profile fitting of CoMOF_new_ as prepared; Table S4. Lattice Parameters derived from Whole Pattern Profile Fitting; Figure S15. (a) Plot of the lengths of the **b** versus **a** axes; (b) the strain tensor for water removal after heating for CoMOF_new_ after treatment at 140 °C for 1 h (PDF)
