# Supplementary material for: Enhanced CO2 Uptake in Cobalt-Based MOFs via Textural Optimization and Water-Induced Flexibility
Source: ACS Appl Mater Interfaces. 2026 Jun 10;18(24):33873–85. doi: 10.1021/acsami.6c04958 (PMC13307064; doi:10.1021/acsami.6c04958)
Supplement: Supplementary file 1 [file am6c04958_si_001.pdf]

Supporting Information

Enhanced CO_2 Uptake in Cobalt-Based MOFs via Textural Optimization and Water-Induced Flexibility

Mariangela Oggianu,[1,2‡] Chiara Busonera,[1,2‡] Fabio Manna,[1,2] Valentina Mameli,[1,2] Francesca Perra,[1,2] Virginia Guiotto,[3] Valentina Crocellà,[3] Luciano Atzori,[1,2] Norberto Masciocchi,[4] Elisabetta Rombi,[1,2] Carla Cannas,[1,2] and Maria Laura Mercuri[1,2*]*

[1] Department of Chemical and Geological Sciences, University of Cagliari, S.S.554 bivio per Sestu, 09042 Monserrato, CA, Italy

[2] Consorzio Interuniversitario Nazionale per la Scienza e Tecnologia dei Materiali (INSTM), Via Giuseppe Giusti 9, 50121 Firenze, FI, Italy

[3] Department of Chemistry, NIS and INSTM Reference Centers, Università di Torino, Turin 10135, Italy.

[4] Dipartimento di Scienza e Alta Tecnologia and To.Sca.Lab, Università degli Studi dell'Insubria, via Valleggio 11, 22100 Como, Italy.

e-mail: ccannas@unica.it, mercuri@unica.it

Summary

Scheme S1. 2
Figure S1. 3
Scheme S2. 3
Figure S2. 4
Figure S3. 4
Supplementary Text, ST1: Pore Analysis 5
Figure S4. 7
Table S1. 7
Scheme S3. 8
Figure S5. 9
Table S2. 9
Table S3. 10

Figure S6. 14
Figure S7. 14
Figure S8. 15
Supplementary Text, ST2: Breakthrough curve Analysis 15
Figure S10. 16
Figure S11. 17
Supplementary Text, ST3: The effect of water elimination and reinsertion 18
Figure S12 18
Figure S13. 18
Figure S14 19
Table S4. 19
Figure S15. 19
References 21

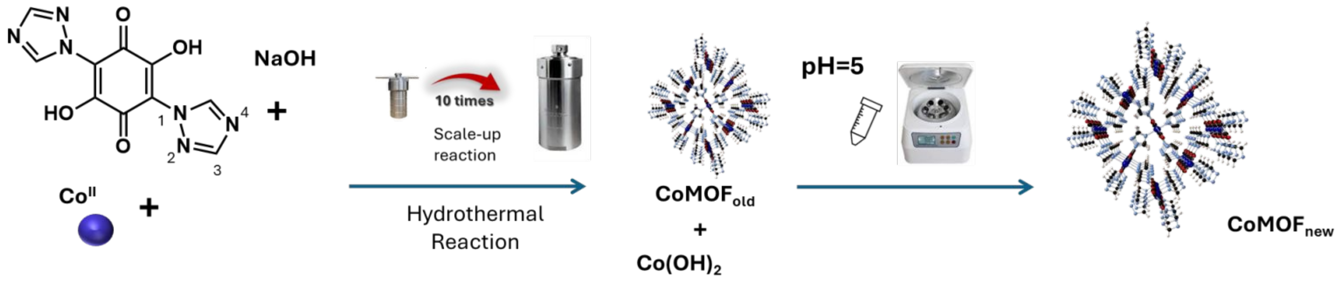

Scheme S1. Post-synthetic purification procedure for CoMOF$_{new}$

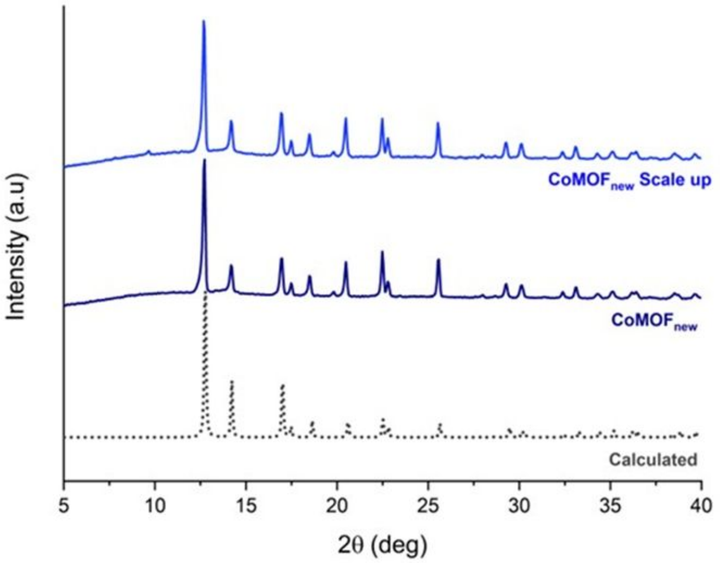

Figure S1. Calculated (grey dashed line) and experimental PXRD patterns of $CoMOF_{new}$ (blue navy line) and $CoMOF_{new}$ at the gram-scale (scale up, blue)

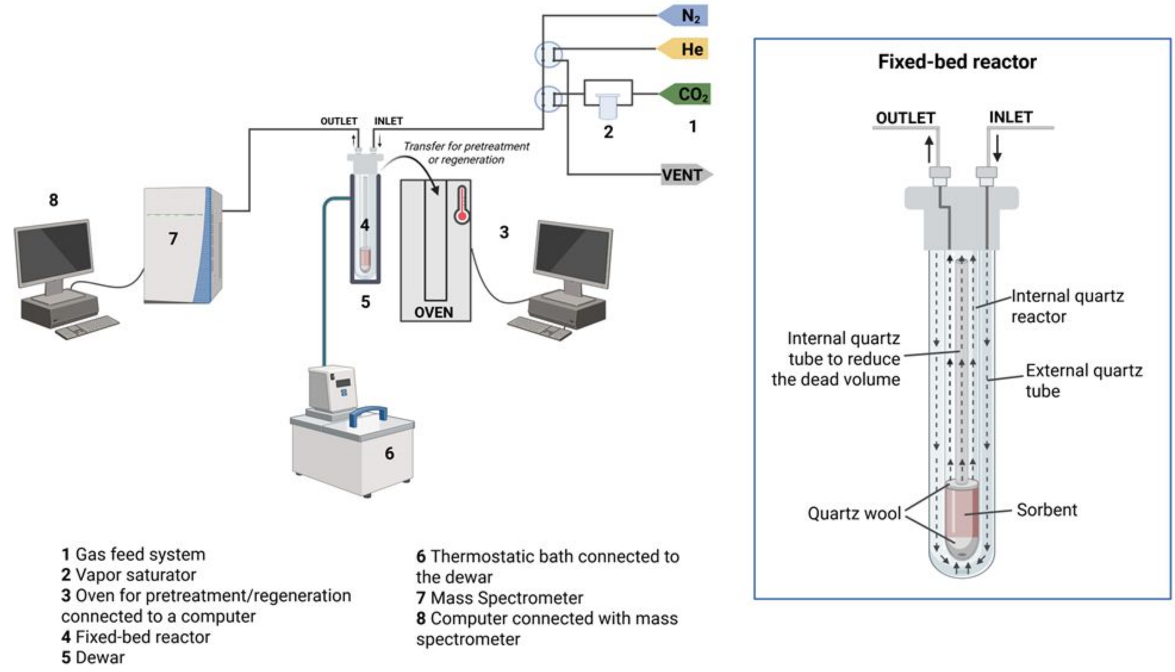

Scheme S2. Schematic representation of the home-made apparatus for breakthrough measurements Created in BioRender. Busonera, C. (2026) https://BioRender.com/qu4tueh

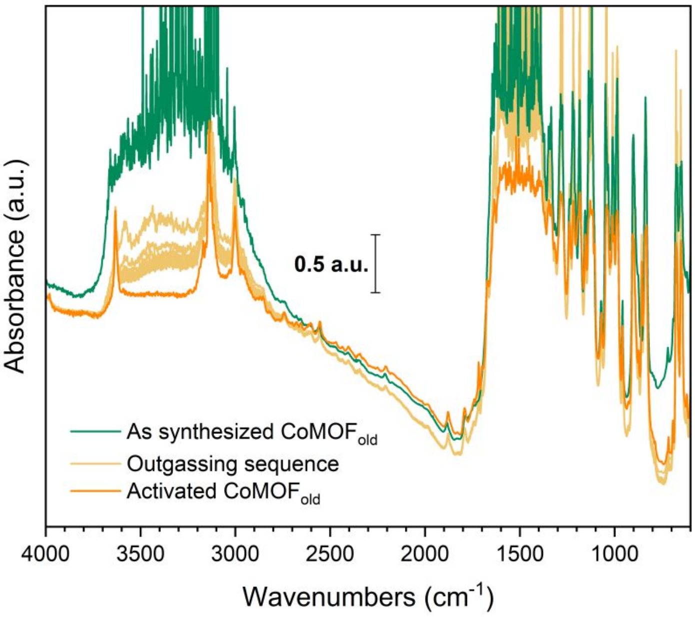

Figure S2. *In situ* FT-IR spectra of as synthesized $CoMOF_{old}$ (green spectrum), during outgassing sequence performed at room temperature (pale orange spectra), and after 12 h at 80°C under vacuum (orange spectrum).

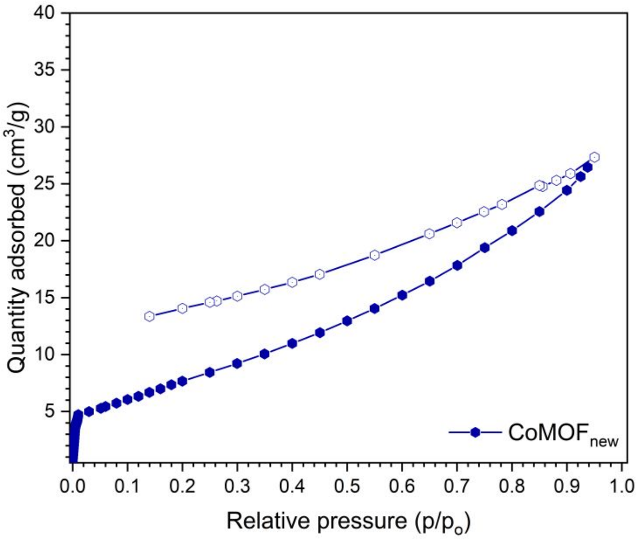

Figure S3. Ar adsorption/desorption isotherm collected on $CoMOF_{new}$ at -183.15 °C.

Supplementary Text, ST1: Pore Analysis

The peanut-shaped cavities can be modeled as elliptical cylinders with a height of 9.06 Å (distance H8···H8), a minor axis *b* of 3.43 Å (corresponding to the maximum pore diameter), and a major axis *a* of 4.72 Å, calculated by taking into account the cavity volume (**Figure S4a**, **Figure 3b**). Considering the cylindrical shape of the pores, and that they have a minor axis *b* that is slightly larger than the kinetic diameter of CO_2 $D_k(CO_2)$ = 3.30 Å, each pore can accommodate up to two CO_2 molecules. CO_2 is a linear molecule whose shape can be modeled by both cylindrical or ellipsoidal representations (see **Figures S4b-c**). The ellipsoidal approximation yields an occupied volume of ~37 Å^3 per CO_2 molecule, smaller than that estimated via the cylindrical model (~46 Å^3). Using the ellipsoidal volume (37 Å^3) and the experimental uptake of 1.34–1.66 molecules per formula unit (corresponding to ~2.7–3.4 molecules per unit cell), the calculated volume occupied by adsorbed CO_2 is estimated at 100-126 Å^3 per unit cell (or 0.089–0.114 cm^3 g^{-1}). This value (0.114 cm^3 g^{-1}) is in close agreement with the pore volume determined *via* NL-DFT analysis (0.120 cm³/g), supporting the validity of the adopted approximation. With this approximation we can hypothesize the arrangement of the two molecules of CO_2 inside the cylindrical cavities as described in **Figure S4a**.

The detailed calculations are reported as follow: the height (*c*) of this cylinder can be approximated to that of the distance H8···H8 (9.056 Å); the semi-minor axis (*b*) of the cylinder can be approximated to half of the maximum pore diameter (3.43 Å), as this is also equivalent to the maximum diameter of the sphere that can fit into the pore. As further confirmation, this value is very similar to that of the intermolecular distance between H8···H8 (4.971 Å), which delimits the pore along this direction, minus twice the Van der Waals radius (r_{VdW}) of H (4.971 Å - 2 × 1.20 Å ≈ 3.57 Å) which we noted to be practically the same value obtained from the Pore Size Distribution (3.6 Å), as further confirmation that this is a good approximation; the length of the other semi-axis (*a*) can be derived from the other parameters as shown in **Figure S4a** and is approximately 2.36 Å. As can be seen in **Figure 3b**, the triazole rings that delimit the pore in this direction lie on two parallel planes that are around 7.90 Å apart, and consequently can also be roughly approximated to this distance minus twice the r_{VdW} of N (7.90 Å – 2 × 1.50 Å= 4.9 Å) or r_{VdW} of C (7.9 Å – 2 × 1.7 Å= 4.5 Å). These values are in accordance with the one derived from the cylinder model (2.36 Å × 2 = 4.72 Å), all the cylinder parameters discussed are also reported in **Figure S4a** and the distances mentioned are highlighted in **Figure 3b**.

In accordance with the r_{VdW} and bond distances, a length of 5.36 Å can be derived ($d_{O\text{-}O}$ =2.32 Å ; 1.52 Å × 2 + 2.32 Å = 5.36 Å), while the diameter can be approximated to that of the kinetic diameter (3.30 Å), which is a value between the d_{VdW} of O (3.04 Å) and C (3.40 Å) that are in some report are approximated to the CO_2 diameter. [1–4] The volume can, also in this case, be approximated to that of a cylinder, although this is an overestimation, as shown in **Figure S4b**. The value thus obtained is approximately 46 Å^3 and gives us a better visualization of the steric hindrance of this linear molecule compared to the kinetic diameter value only, despite this is often used in the gas-sorption context for comparison.[5] Furthermore, this evidences that the volume of two CO_2 molecules is smaller than that of the pore, which is therefore large enough to accommodate them. A better approximation is shown in **Figure S4c**, considering the thermodynamic parameters of CO_2 often used in absorption models and the shape of the molecule as an ellipsoid. In this case, the minor-axis is considered equal to the

kinetic diameter (3.30 Å) and the perpendicular cross-sectional area equivalent to that of CO_2 at 196 K (17.0 Å^2). [5]Therefore, a molecular volume of 37 Å^3 is obtained.

For comparison purposes, we calculated also the porosity parameters of two ultramicroporous MOFs, CALF-20,[6] which is considered one of the most promising materials for CO_2 capture, and $Cu_4I_4[(C_{34}H_{30}N_8)$,[7] which simirarly to the Co-MOF shows isolated voids, using the same parameter and we compared it in **Table S1**. As can be seen, all three MOFs show a null network-accessible surface area and a pore limiting diameter below 3.30 Å (kinetic diameter of CO_2), so in principle CO_2 should not diffuse into the pores analogously. For example, in the case of CALF-20, it has recently been demonstrated that adsorbed guests clearly increase the pore space for more adsorbates thanks to the flexibility of the MOF which trigger a phase transition, in this case there is a global expansion of the cell following the interaction with CO_2 favouring the diffusion inside the pores.[8] The case of $Cu_4I_4[(C_{34}H_{30}N_8)$ is different. Here, the MOF has distinct cavities rather than open 3D channels, like our case, and through a mechanism called "Magic door", the pores can open thanks to local structural changes in the framework and allow CO_2 molecules to pass preferentially through, instead of the N_2 molecules, due to greater interaction and lower activation energy (E_a) during the diffusion process. This framework has the property of trapping CO_2 inside the cavities and keeping it kinetically locked inside for long periods of time, as it requires adequate activation energy to open the pores. This leads to highly selective adsorption governed by small differences in E_a and/or adsorption enthalpy, with low adsorption heats and therefore a low energy regeneration. [7] This scenario is very similar to our situation: in fact, a small rotation of the triazole rings can lead to the formation of pore aperture between the cavities suitable for the passage of CO_2 (d > 3.30 Å) in the Co-MOF. It is important to note that these two mechanisms are the two extreme cases, as shown in the works of Kitagawa et al., and the two and other mechanism can coexist within the same framework but also be dependent on pressure, temperature and adsorbate molecule.[1,4] For completeness, we have included the energy diagrams for the different mechanisms, according to the work of Shimada et al.[7], in **Scheme S3**, together with a sketch of the suggested mechanism in accordance with the calculated and experimental parameters and literature reports. [1,4,7]The associated activation energy is expected to be relatively low compared to the experimental enthalpy of CO_2 adsorption (~20 kcal mol^{-1}) and can be reasonably estimated to lie below 10 kcal mol^{-1}, consistent with reported hydrogen bond strengths in 1,2,4-triazole systems. [9,10]

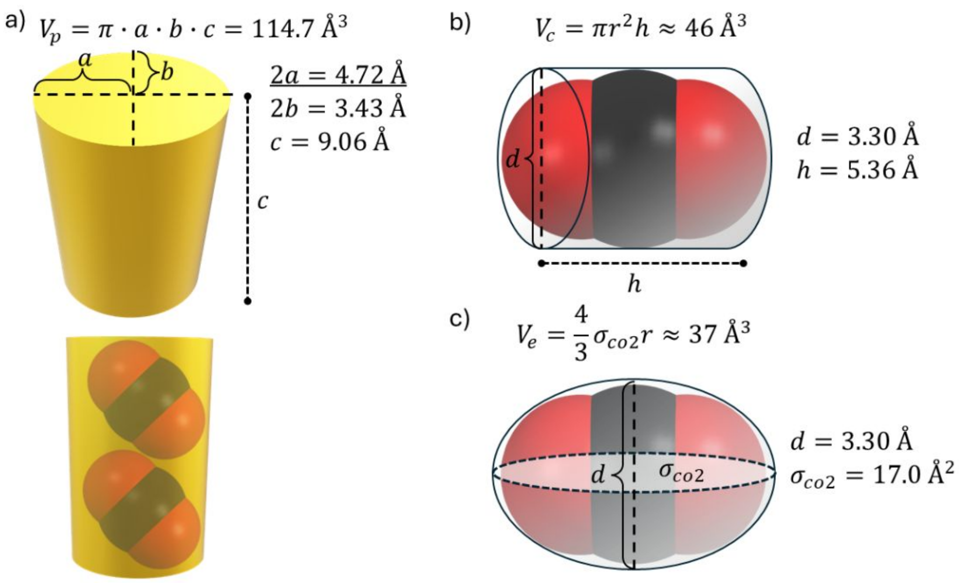

Figure S4. a) the volume of a single cavity approximated as a cylinder where: V_p is derived from void volume (probe radius: 1.2 Å; grid spacing: 0.3 Å); b is equal to half the pore limiting diameter; c is equal to H8···H8 distance (see text) and a is derived from the other (top). Oversimplification of the two CO_2 inside a single cylindrical cavity (bottom). b) CO_2 volume and parameter for one molecule with shape approximated as a cylinder where: d is equal to the CO_2 kinetic diameter and h to the CO_2 length with Van der Waals radii approximation (see text). c) CO_2 volume and parameter for one molecule with shape approximated as a ellipsoid according to the thermodynamic parameters (d = kinetic diameter; s_{CO2} = cross-sectional area at 196 K).

Table S1. The pore parameters calculated for Co(trz_2An), CALF-20 & $Cu_4I_4[(C_{34}H_{30}N_8)$

Parameter	Co(trz_2An)[11]	CALF-20[6]	$Cu_4I_4[(C_{34}H_{30}N_8)$[7]	Unit
System Volume	764.900	737.164	4422.042	Å³
System Mass	662.219	709.903	5274.014	g/mol
System Density	1.438	1.599	1.980	g/cm³
Void Volume[a]	30.0	40.5	17.7	%
Total surface area	0	0	12.09	Å²
Total surface area per volume	0	0	27.34	m²/cm³
Total surface area per mass	0	0	13.80	m²/g
Network-accessible surface area	0	0	0.00	Å²
Network-accessible surface area per volume	0	0	0.00	m²/cm³
Network-accessible surface area per mass	0	0	0.00	m²/g
Total helium volume	206.984	258.324	903.876	Å³
Total helium volume	0.188	0.219	0.103	cm³/g
Total geometric volume	345.955	363.108	1.694.907	Å³
Total geometric volume	0.315	0.308	0.194	cm³/g

Network-accessible helium volume	206.984	258.324	903.856	Å³
Network-accessible helium volume	0.188	0.219	0.103	cm³/g
Network-accessible geometric volume	345.892	362.913	1.693.336	Å³
Network-accessible geometric volume	0.315	0.308	0.193	cm³/g
Pore limiting diameter	1.72	2.63	1.30	Å
Maximum pore diameter	3.43	4.13	4.25	Å
[a] probe radius: 1.2 Å; grid spacing: 0.3 Å.				

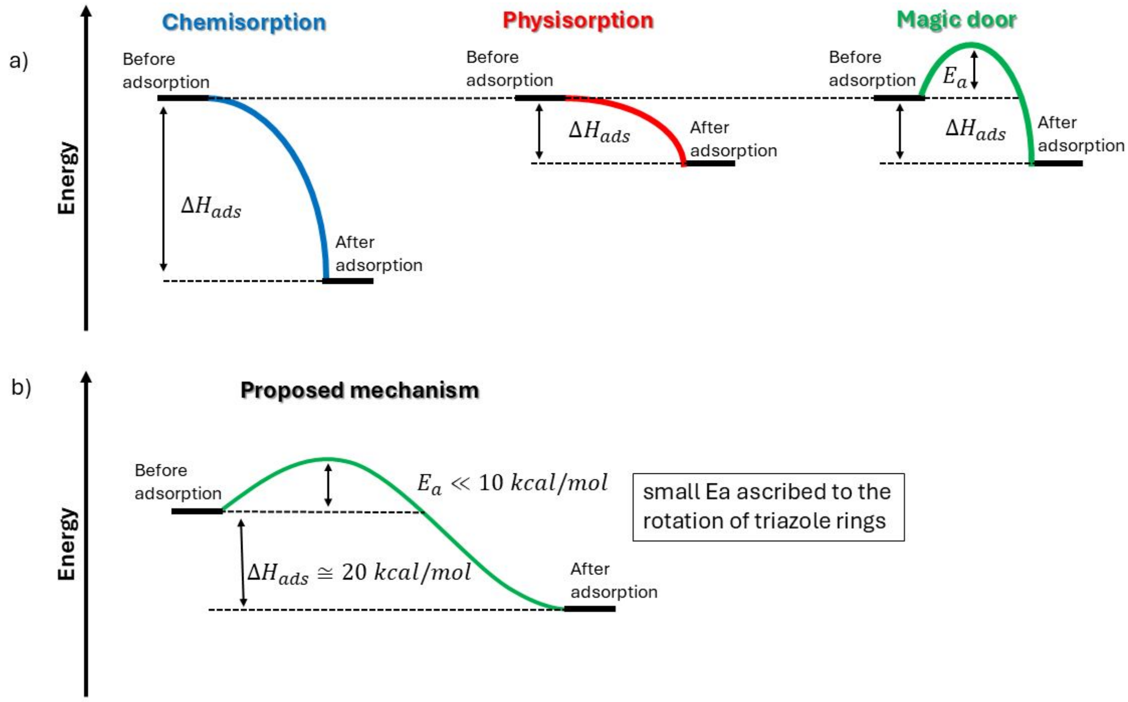

Scheme S3. a) Comparison between the adsorption energy diagrams for chemisorption, physisorption, and "magic door" mechanisms according to Shimada et al.[7] b) Proposed mechanism for CoMOF according to the experimental findings and literature report (see text).

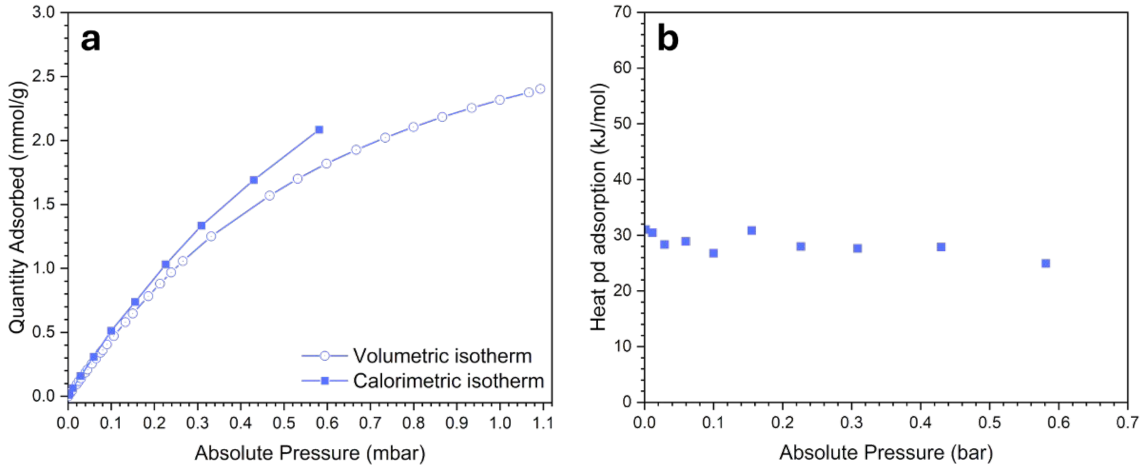

Figure S5. a) CO_2 adsorption isotherms collected on $CoMOF_{new}$ at 40 °C using an ASAP2020 sorption analyzer (empty circles) and a microcalorimetric apparatus (full squares). b) differential molar heat of adsorption.

Table S2. Values of the parameters obtained by fitting the volumetric isotherms of $CoMOF_{new}$ with the Freundlich-Langmuir (FL).

Sample	a_1 (mmol g^{-1})	b_1 (bar^{-c})	c^*	a_{tot}^{**} (mmol g^{-1})	R^2
**$CoMOF_{new}$**	8.35±0.57	1.14±0.15	0.99±0.03	4.45±0.30	0.99976

*heterogeneity exponent of the FL equation; **CO_2 uptake at P = 1 bar

Table S3. State of art for MOFs for CO_2 capture: Linker, node, textural properties, static and dynamic CO_2 uptake

Sample	Linker & Metal Node	C (m^2 g^{-1}) Pore Size (Å)	CO_2 Ads ($mmol\ g^{-1}$) in static	T (°C) P (bar)	Q (KJ mol^{-1})	Gas Mixture Flow (ml min^{-1}) T (°C)	CO_2 Ads ($mmol\ g^{-1}$) in dry dynamic	CO_2 Ads ($mmol\ g^{-1}$) in wet dynamic	Selectivity IAST/a	Setup	Cycles	Regeneration	Ref
**$CoMOF_{new}$**	**$Traz_2An$ Co (II)**	**617 [a]**	**5.00**	**0 1**	**32→ 17**	**5:95 CO_2:N_2 15, 25 10:90 CO_2:N_2 15, 30**	**0.48 0-71**	**1.17**		***Dynamic Experiments*: TPD - MS (Pro Lab Thermo-electron corporation**	**15**	**15 ml min^{-1} N_2 flow for 1.5 h**	**This Work**
$CoMOF_{old}$	$Traz_2An$ Co (II)	431 [a] 3.43	3.80	0 1	25→ 21[f]	5:95 CO_2:N_2 15, 25	0.33		>1000 a 5% CO2	ABR (HIDEN Isochema) instrument	40	40 **ml min^{-1}**Ar flow for 20 minutes	11
Mg-MOF74	DOBDC Mg (II)	1492 [b] 11.0	8.24	25 1	42[f]	15:85 CO_2:N_2 10, 25	2.45	6.7 in static conditions RH20 %	148 IAST 20 %	Homemade apparatus	--	--	14,15
Co_MOF74	DOBDC Co (II)	1404 [b] 8.32	3.67	25 1.1	34 [f]	20:80 CO_2:N_2 5, 25 °C	1.97		--	Homemade apparatus	--	--	16
Ni_MOF74	DOBDC Ni (II)	1418 [b] 8.54	4.06	25 1.1	39 [f]	20:80 CO_2:N_2 5 , 25	1.88		49 IAST 20 %		--	--	16
MIL -160	FDCA Al(OH)(CH_3COO)$_2$	911 [b] 15.36	4.30	20 1	37[f]	15.4:84.6 CO_2:N_2 5, 25	1.41		31.5 IAST 15 % CO_2	Homemade apparatus		80 °C for 12 h	17,18
IISERP-MOF2	4PyC Ni (II)	505 [b] 4.7	4.00	30 1.18	33[e]	14:86 CO_2:N_2 1,-	3.97	3.68	1853 IAST 14% CO_2	homemade apparatus	2	He flow for 20 min	19
MUV-a26	HINA	267 [a]	2.80	10	34.8 → 31.4 [f]	50:50 CO_2:N_2	0.81	--	>1000 a	ABR (HIDEN Isochema) automated	10	40 ml min^{-1} Ar flow for 20 min	20

	[$Fe_3O(CH_3COO)_6$]<br>$ClO_4 \cdot 3H_2O$<br>cluster	3.58		6		15 , 25			5% CO_2	breakthrough analyser setup			
CALF-20	1,2,4-Trz, Ox<br>Zn (II)	528 [b]<br>4.13	4.10	20<br>1.2	40	5:95 CO_2:N_2<br>--, 23.4 °C	1.50	2.59<br>20 % CO_2	230 IAST<br>5% CO_2	dynamic column breakthrough (DCB) apparatus	2	He flow for 12 h at 150°C	6,21
UTSA-120	DPT<br>Cu (II)	638 [b]<br>4.6	5.00	23<br>1	27 → 31	15:85 CO_2:N_2<br>2, --	--		600 IAST<br>15% CO_2		3	20 ml min^{-1} He flow for 15 min at 298 K	22
MIL-120(Al)-AP	BTeC<br>Al (III)	590 [b]<br>6.5	1.90	25<br>1	41→ 36<br>[f]	15:85 CO_2:N_2<br>--	--		>80 IAST<br>15% CO_2	homemade apparatus	6	33 ml min^{-1} N_2 flow 30 °C	23
JLU-MOF56	BTBA<br>Ni (II)	334 [d]<br>3.5	1.06	25<br>1	32.6	5:95 CO_2:N_2<br>--,--	--		32.8 IAST	Micromeritics ASAP 2020 and Micromeritics 3-Flex instruments	--	--	24
mCB-MOF-1	mCB-L<br>$Cu_2(OOC)_4$	756 [b]<br>7	1.40	23<br>1	24.7 → 25.3 [f]	5:95 CO_2:N_2<br>15, 25	0.09	0.09	26.8<br>5% CO_2	ABR (HIDEN Isochema) instrument	2	15 ml min^{-1} Ar flow for 20min at 25°C	12,25
$Fe_2(BDP)_3$	H_2BDP<br>Fe(III)	1230 [b]<br>4.9	3.10	25<br>1		15:85 CO_2:N_2<br>20, 30	0.80		22 (alfa) 298K<br>15% CO_2	Bronkhorst mass flow controllers	11	20 ml min^{-1} He flow for 20 min at 120°C	26,27
SIFSIX-3-Cu	Pyrazine<br>Cu(II)	300 [c]<br>3.5	2.50	25<br>1	54<br>[f]	--<br>--<br>--	--		10500<br>1p.p.m		4	5 ml min^{-1} He flow at 50°C	28
Cu-F-pymo	F-pymo<br>Cu (II)	146 [d]<br>3.6	3.09	25<br>1	29.1 near-zero coverage<br>[f]	15:85 CO_2:N_2<br>6	0.94		< 10^7<br>15% CO_2	homemade apparatus	5	60 ml min^{-1} He flow for 20 min at 25°C	29
MOF303-Al	PDC	1529 [b]	5.1		25.2	10000ppm	0.36	0.34	15	SIRIUS-XF-30 instrument equipped with a	50		30

	Al(III)	5.9				20, 25		RH 50 %	50%CO_2: N_2<br>4<br>50%CO_2: CH_4	GC 9790II gas chromatograph			
MgCUK-1	2,4-PCD<br>Mg^{II}	600 [d]	3					3.3<br>In static conditions<br>RH 20%					31
Ni-TM DMOF	TM-BDC<br>Ni^{II}	829 [b]	5	0<br>1	27 [f]	CO_2:N_2mixture (15/85, v/v),<br>10, 25	0.88	0.83<br>80 % RH	33 IAST	Micromeritics Breakthrough Analyzer (BTA)			32
TAMOF-1	*S*–HTA<br>Cu (II)	980[b]<br>10	3.80	16<br>1	26- 27 [f]	50:50 CO_2:CH_4<br>18.7, --	2.71	2.31<br>RH 72%	35.6 (IAST)<br>30% CO_2			N_2 flow at 25°C	33
MUF-16	Haip<br>Co (II)	214[b]<br>4.6	2.13	15<br>1	32 → 49 [f]	50:50 CO_2:CH_4<br>6, 23	1.20		6690 IAST<br>50% CO_2		12	5 ml min^{-1} inert gas for 18 min at 20 °C then 80 °C for 20 min	34
Cu-MOF	H_2hfipbb<br>Cu (II)	105 [a]		25<br>1	28.3→27.9 [g]	15:85 CO_2:N_2<br>2	--		6.9 a<br>15 % CO_2				35
Qc-5-M-sql B	HQc<br>Cu (II)	488 [d]<br>4.8	1.56	15<br>1	36<br>[f]	15:85 CO_2:N_2<br>2<br>--,--	1.98	1.85	19 (IAST)<br>15 % CO_2			44 ml min^{-1} flow of He gas at 100 °C	36
CuMOF	$Traz_2An$<br>Cu (II)	410 [a]<br>3.4	2.09		27.2	50:50 CO_2:N_2<br>15, 25	1.62	1.78	80 IAST<br>20%	Homemade apparatus	5		37

[a] CO_2 at 273 K; [b] N_2 at 77 K; [c] CO_2 at 298 K; [d] CO_2 at 195 K; [e] DFT fClaius-Clapeyron [g]van't Hoff equation. DOBDC :1,5-dioxido-1,4-benzenedicarboxylate); FDCA: 2,5-furandicarboxylic acid; 4PyC: 4-pyridine carboxylic acid; HINA: isonicotinic acid; 1,2,4-Trz: 1,2,4-triazolate, Ox: oxalate; mCB-L: 1,7-di(4-carboxyphenyl)-1,7-dicarba-closo-dodecaborane; DPT: 3,6-di(4-pyridyl)-1,2,4,5-tetrazine; BTeC: 1,2,4,5-benzenetetracarboxylic acid; BTBA: 3,5-bis(triazol-1-yl)benzoic acid); H_2BDP: 1,4-benzenedipyrazole; F-pymo: 5-fluoro-2-hydroxypyrimidine; PDC: 3,5-Pyrazoledicarboxylic acid monohydrate; *S*–HTA : imidazole-5-ylmethyl -(1,2,4–triazol-4-yl)acetate; Haip: 5-aminoisophthalic acid: H_2hfipbb: 4,4′-(hexafluoroisopropylidene)-bis benzoic acid; HQc: quinoline-5-carboxylic acid; Traz$_2$An

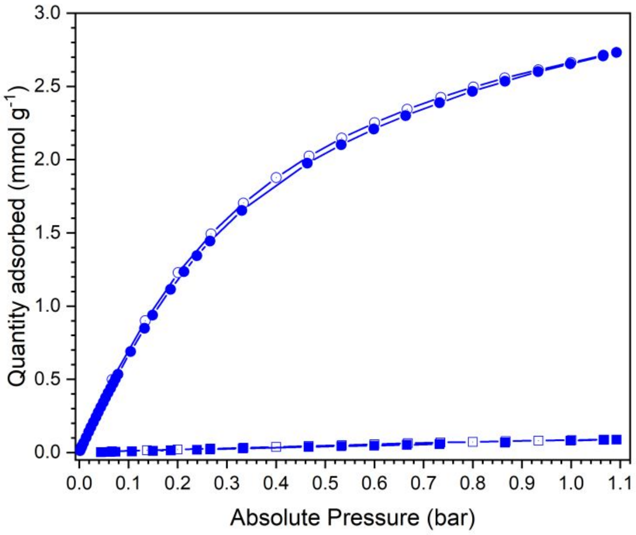

Figure S6. CO_2 (circles) and N_2 (squares) adsorption/desorption isotherms collected on $CoMOF_{new}$ at 30°C.

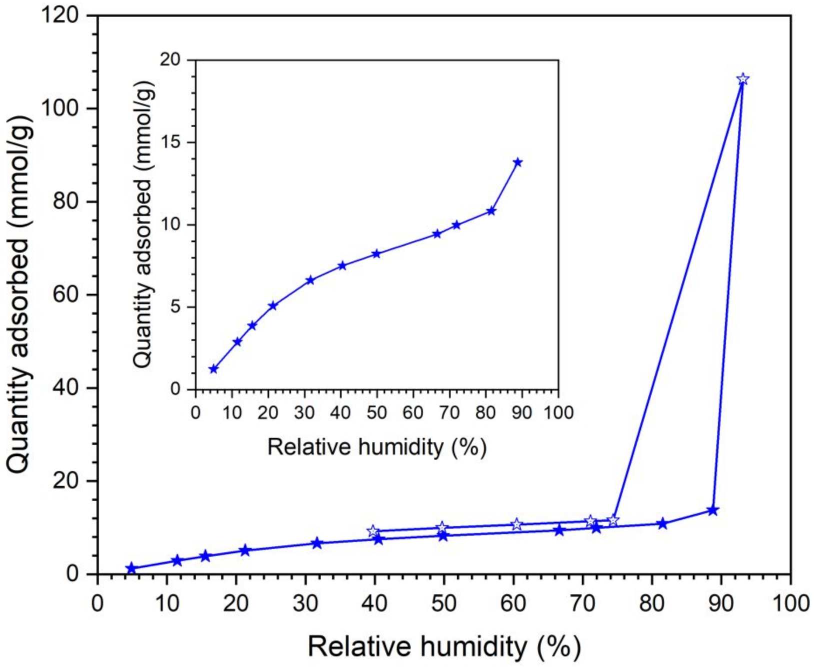

Figure S7. H_2O adsorption/desorption isotherms collected on $CoMOF_{new}$ at 30°C. The inset graph displays the isotherm up to 90% RH, namely before water condensation.

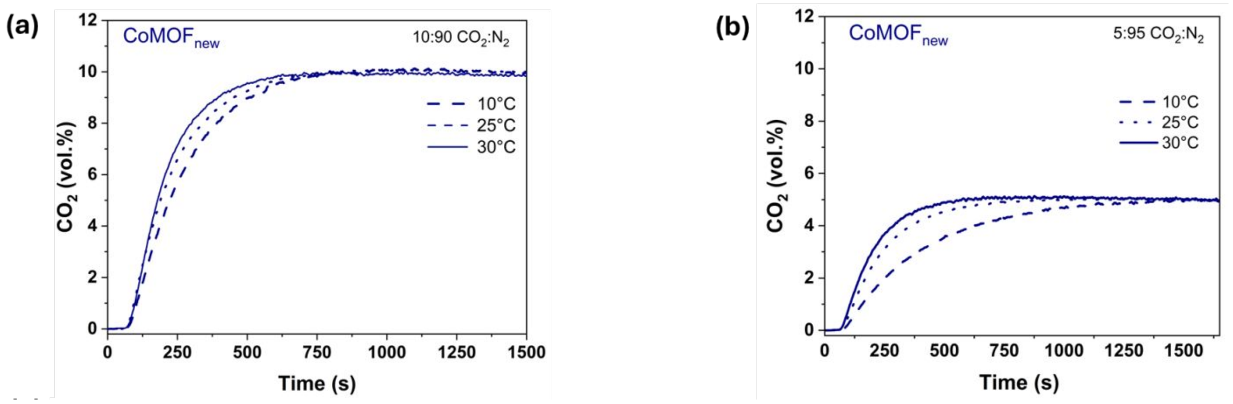

Figure S8. Breakthrough curves acquired at different temperatures and CO_2 inlet concentrations for $CoMOF_{new}$ (a,b).

Supplementary Text, ST2: Breakthrough curve Analysis

Breakthrough curves are influenced by several factors, including the adsorption capacity of the material, the total flow rate of the gas mixture, and the initial concentration of the adsorbate. Additional parameters such as temperature, bed height, particle size, and mass transfer resistance also play a significant role in determining the shape and position of the curves.

The breakthrough time is conventionally defined as the moment at which the leading edge of the mass transfer zone reaches the outlet of the adsorbent bed, resulting in an appreciable outlet concentration, typically corresponding to 5% ($t_{5\%}$) of the inlet CO_2 concentration[12]. This characteristic time depends on several factors, including thermodynamic adsorption capacity, adsorption kinetics, and mass-transfer rates.

With 10% CO_2 in the feed (**Figure S8a**), $t_{5\%}$ was found to be practically independent of temperature (85-92 s), suggesting that the initial increase in outlet CO_2 concentration is mainly governed by interparticle (external) mass-transfer effects rather than by adsorption within the MOF pores. A similar behavior was observed using the feed containing 5% CO_2 (**Figure S8b**), where $t_{5\%}$ was also almost temperature-independent (80-100 s) and comparable to that measured at higher inlet CO_2 concentration. This finding confirms that, in the initial portion of the breakthrough curves, transport is primarily controlled by the feed flow rate rather than by adsorption phenomena.

These observations indicate that the precise identification of an intrinsic breakthrough time associated with adsorption within the porous structure can be challenging under these conditions, thereby complicating a rigorous interpretation of the mass transfer zone, which is typically identified between $t_{5\%}$ ($C_{out}/ C_{in}= 0.05$) and $t_{95\%}$ ($C_{out}/ C_{in}= 0.95$)[13].

At both CO_2 concentrations, the effect of temperature becomes clearly visible in the later portion of the breakthrough curves, where CO_2 uptake evolves more consistently with intraparticle mass transport within the MOF pores, where adsorption predominantly occurs.

For the 10% CO_2 feed, since the shape of the breakthrough curves is only weakly affected by temperature, the increase in dynamic uptake observed at lower temperatures can mainly be attributed to the enhanced adsorption capacity of the sorbent, although a possible contribution from increased diffusional limitations cannot be excluded. In this context, the markedly different shape of the breakthrough curve obtained with the 5% CO_2 feed at 10 °C, compared with those measured at 25 °C and 30 °C, suggests a stronger influence of diffusional limitations, as expected at low CO_2 partial pressure and temperature.

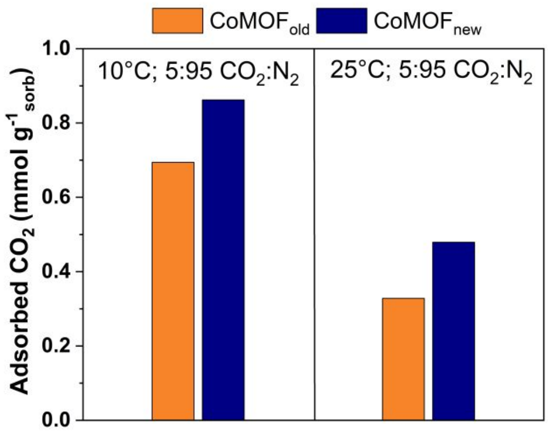

Figure S9. Comparison of CO_2 uptake on $CoMOF_{old}$ (orange) and $CoMOF_{new}$ (blue) for the 5:95 CO_2:N_2 mixture at 10 °C and 25 °C

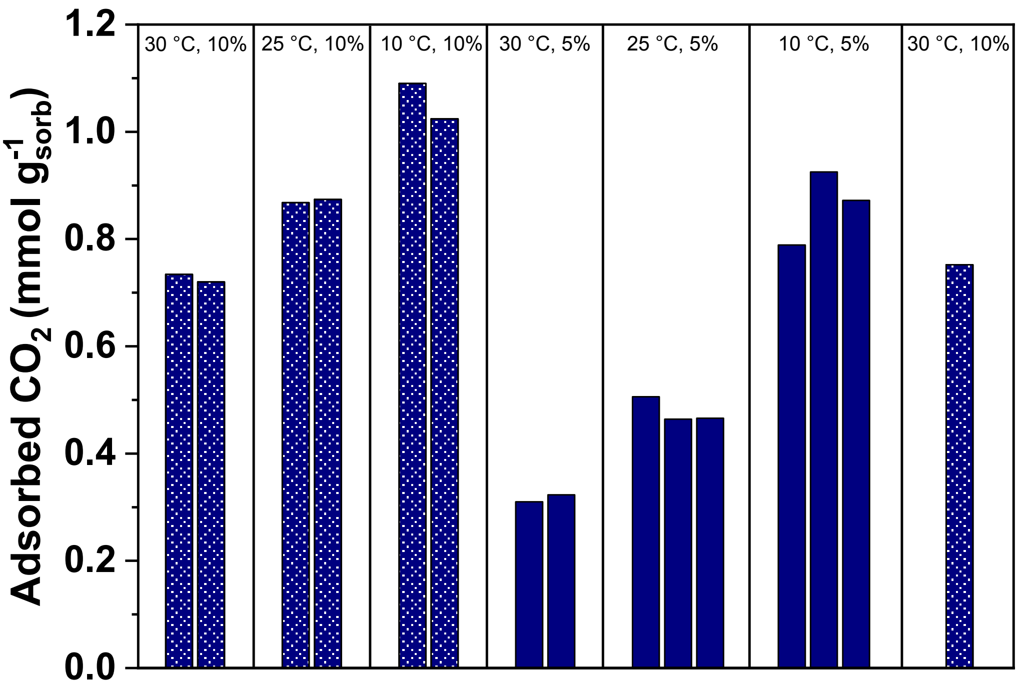

Figure S10. Cyclic stability of $CoMOF_{new}$ evaluated over 15 regeneration cycles.

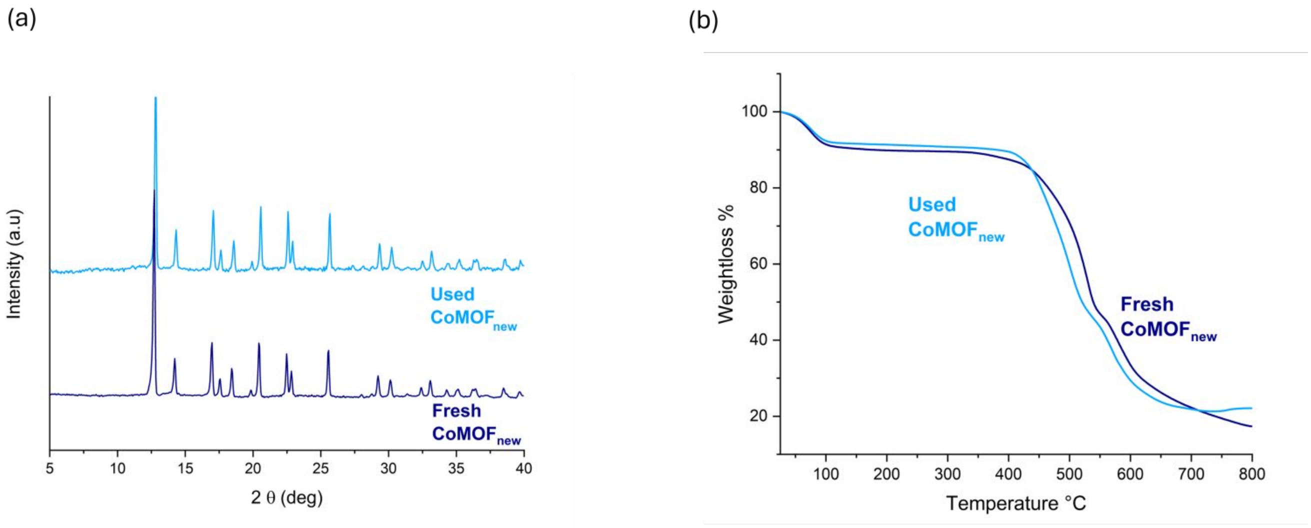

Figure S11. Pattern WA-PXRD (a) and thermogram (b) of $CoMOF_{new}$ before Dynamic Adsorption Test (blue line, fresh) and after 15 adsorption-desorption cycles (light blue line, used)

Supplementary Text, ST3: The effect of water elimination and reinsertion

At least metrically, one expects that *the crystal structure should be different before and after activation*. Regrettably (and also to our surprise), it is not so. The water molecules in the crystal structure our Co-based MOF have no clear structural effect. In order to substantiate this statement, we have performed X-ray Powder Diffraction (XRD) analysis on a sample stored in air, at RT and RH values near 55% (average value in our lab) for two weeks after precipitation (Sample 1), then on the same sample after heating it in air at 140 °C for 1 h in an oven (Sample 2) and on the same (heated) sample exposed to saturated water environment in a closed box for 14 h (Sample 3). Given the above treatments, we expected that the hydration levels were decreasing in the 3, 1, 2 order.

Figure S12 shows that the XRD patterns are nearly superimposable (though some very minor differences exist, later discussed).

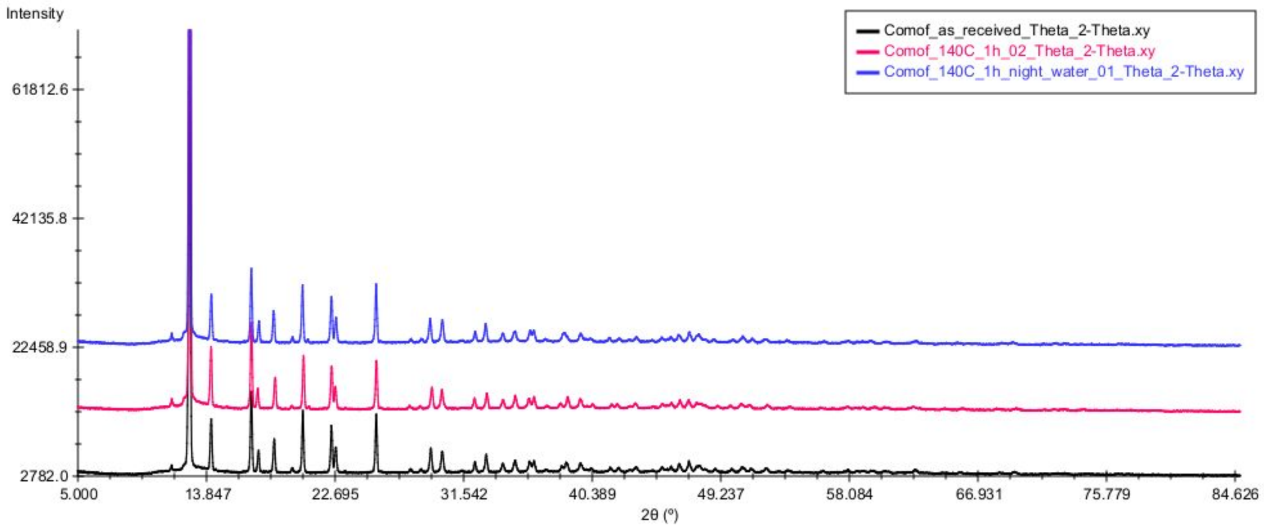

Figure S12. Raw data for Sample 1 (black), Sample 2 (dehydrated, red) and Sample 3 (rehydrated, blue).

This speaks for a significant rigidity of the Co-MOF framework, in that crystal periodicity, space group symmetry and framework structure are kept constant throughout the (de)hydration process. However, on expanding a limited portion of the XRD traces, some minor differences appear (see **Figure S13**).

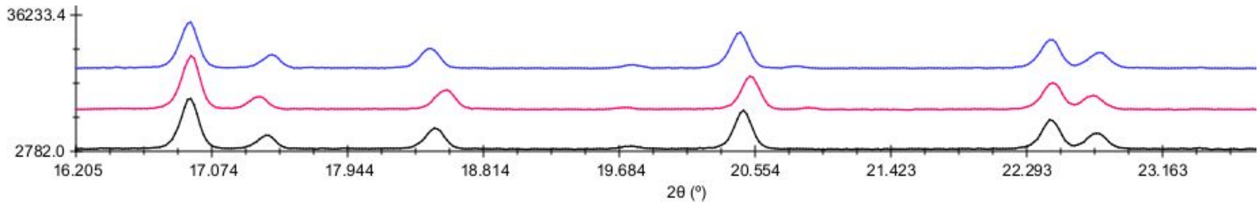

Figure S13. Raw XRPD data for Sample 1 (black), Sample 2 (dehydrated, red) and Sample 3 (rehydrated, blue), plotted in the limited range; small shifts attributed to the countercorrelated changes of the **a** and **b** axes are observed and quantified by the Pawley-type analysis exemplified in **Figure S14**.

These differences were interpreted through the aid of Pawley-type fitting, an example of which is graphically presented in **Figure S14**.

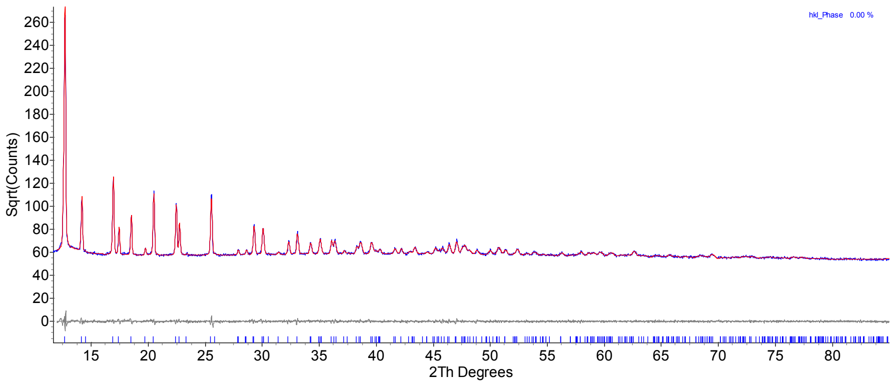

Figure S14. Pawley-type full profile fitting Sample 1: observed data in blue, simulated pattern in red; the difference plot (in grey) and the peak markers (in blue) are shown at the bottom. Note that, in order to enhance the intensities of the weak peaks, the square-root of counts is used in the y axis.

The derived cell parameters are gathered in **Table S4** below.

Table S4. Lattice Parameters derived from Whole Pattern Profile Fitting

Sample	System	a, Å	b, Å	c, Å	V, Å^3
Sample 1	Orthorhombic	10.19	9.60	7.93	776
Sample 2	Orthorhombic	10.23	9.57	7.94	777
Sample 3	Orthorhombic	10.18	9.63	7.94	778

These values confirm the absence of evident structural (ordered) water molecules, since a single H_2O moiety accounts for ca. 25-30 Å^3; this makes the hydrated material a nice example of channel hydrates, often non-stoichiometric, which have found large applications in the pharmaceutical field.

A plot of the *b* vs *a* variability (see **Figure S15**, the length of the **c** axis being roughly constant) provides a clear countercorrelated effect and indicates a constancy of the molar volume and of the size of the rectangular **ab** mesh. These changes, as anticipated, are very small (maximum deviation < 0.3%).

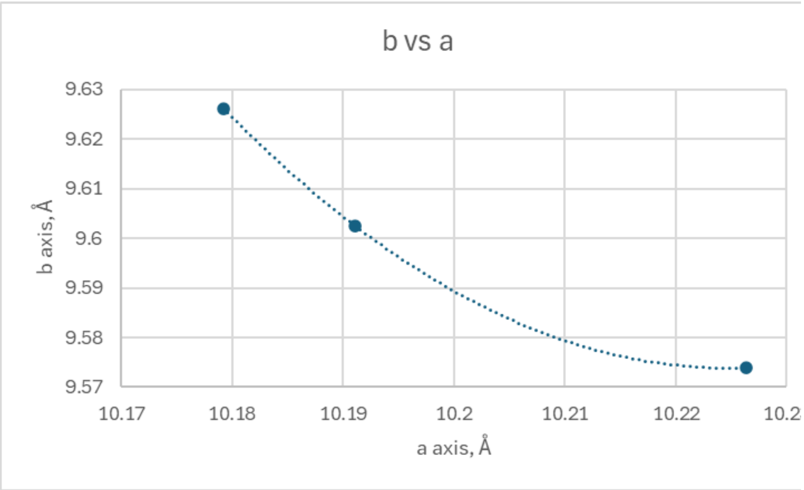

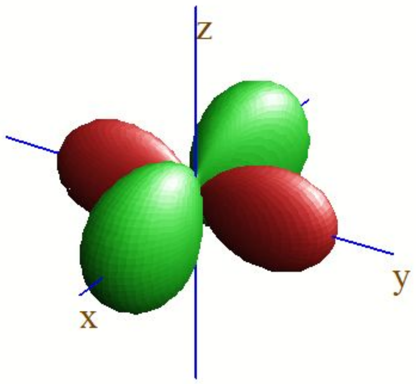

a) b)

Figure S15. a) the lengths of the **a** and **b** axes are countercorrelated; b) the strain tensor for water removal after heating Sample 1 in an oven at 140°C for 1 h. Red means shrinking; Green means expanding. The strain tensor calculation and its graphical representation were made possible by Ohashi's and Kaminski's protocols: Y. Ohashi, A program to calculate the strain tensor from two sets of unit-cell parameters. In Comparative Crystal Chemistry, [38] *Hazen, R.M., Finger, L.W., Eds.; Wiley: Chichester, UK, 1982; pp. 92–102; https://cryst.ehu.es/cryst/strain.html, accessed May*

2026; W. Kaminski, Wintensor V.1.4, 2024: Tensor-drawing and calculation tool for Windows. http://cad4.cpac.washington.edu/WinTensorhome/ WinTensor.htm

Summarizing, there is no significant structural change for this material upon insertion or depletion of water molecules. This static picture can be however changed if dynamics are taken into account, an effect that diffraction methods alone cannot perceive.

References

(1) Gu, Y.; Zheng, J.-J.; Otake, K.; Sakaki, S.; Ashitani, H.; Kubota, Y.; Kawaguchi, S.; Yao, M.-S.; Wang, P.; Wang, Y.; Li, F.; Kitagawa, S. Soft Corrugated Channel with Synergistic Exclusive Discrimination Gating for CO2 Recognition in Gas Mixture. *Nat. Commun.* **2023**, *14* (1), 4245. https://doi.org/10.1038/s41467-023-39470-w.

(2) Geng, S.; Xu, H.; Cao, C.; Pham, T.; Zhao, B.; Zhang, Z. Bioinspired Design of a Giant [Mn 86] Nanocage-Based Metal-Organic Framework with Specific CO 2 Binding Pockets for Highly Selective CO 2 Separation. *Angew. Chemie Int. Ed.* **2023**, *62* (32). https://doi.org/10.1002/anie.202305390.

(3) Kunze, S.; Groll, R.; Besser, B.; Thöming, J. Molecular Diameters of Rarefied Gases. *Sci. Rep.* **2022**, *12* (1), 2057. https://doi.org/10.1038/s41598-022-05871-y.

(4) Sakamoto, H.; Otake, K.; Kitagawa, S. Progressive Gas Adsorption Squeezing through the Narrow Channel of a Soft Porous Crystal of [Co2(4,4′-Bipyridine)3(NO3)4]. *Commun. Mater.* **2024**, *5* (1), 171. https://doi.org/10.1038/s43246-024-00609-x.

(5) Mohsenpour Tehrani, M.; Chehrazi, E. Metal–Organic-Frameworks Based Mixed-Matrix Membranes for CO 2 Separation: An Applicable-Conceptual Approach. *ACS Appl. Mater. Interfaces* **2024**, *16* (26), 32906–32929. https://doi.org/10.1021/acsami.4c06914.

(6) Lin, J.-B.; Nguyen, T. T. T.; Vaidhyanathan, R.; Burner, J.; Taylor, J. M.; Durekova, H.; Akhtar, F.; Mah, R. K.; Ghaffari-Nik, O.; Marx, S.; Fylstra, N.; Iremonger, S. S.; Dawson, K. W.; Sarkar, P.; Hovington, P.; Rajendran, A.; Woo, T. K.; Shimizu, G. K. H. A Scalable Metal-Organic Framework as a Durable Physisorbent for Carbon Dioxide Capture. *Science (80-.).* **2021**, *374* (6574), 1464–1469. https://doi.org/10.1126/science.abi7281.

(7) Shimada, T.; Usov, P. M.; Wada, Y.; Ohtsu, H.; Watanabe, T.; Adachi, K.; Hashizume, D.; Matsumoto, T.; Kawano, M. Long Time CO 2 Storage Under Ambient Conditions in Isolated Voids of a Porous Coordination Network Facilitated by the "Magic Door" Mechanism. *Adv. Sci.* **2024**, *11* (2). https://doi.org/10.1002/advs.202307417.

(8) Oktavian, R.; Goeminne, R.; Glasby, L. T.; Song, P.; Huynh, R.; Qazvini, O. T.; Ghaffari-Nik, O.; Masoumifard, N.; Cordiner, J. L.; Hovington, P.; Van Speybroeck, V.; Moghadam, P. Z. Gas Adsorption and Framework Flexibility of CALF-20 Explored via Experiments and Simulations. *Nat. Commun.* **2024**, *15* (1), 3898. https://doi.org/10.1038/s41467-024-48136-0.

(9) Khodiev, M.; Holikulov, U.; Jumabaev, A.; ISSAOUI, N.; Nikolay Lvovich, L.; Al-Dossary, O. M.; Bousiakoug, L. G. Solvent Effect on the Self-Association of the 1,2,4-Triazole: A DFT Study. *J. Mol. Liq.* **2023**, *382*, 121960. https://doi.org/10.1016/j.molliq.2023.121960.

(10) Mulloev, N. U.; Khodiev, M. K.; Islomov, Z. Z.; Lavrik, N. L. Influence of the Structure of Triazole Derivative Molecules on the Efficiency of Formation of Intermolecular H-Bonds. *J. Struct. Chem.* **2020**, *61* (2), 232–237. https://doi.org/10.1134/S0022476620020079.

(11) Monni, N.; Andres-Garcia, E.; Caamaño, K.; García-López, V.; Clemente-Juan, J. M.; Giménez-Marqués, M.; Oggianu, M.; Cadoni, E.; Mínguez Espallargas, G.; Clemente-León, M.; Mercuri, M. L.; Coronado, E. A Thermally/Chemically Robust and Easily Regenerable Anilato-Based Ultramicroporous 3D MOF for CO 2 Uptake and Separation. *J. Mater. Chem. A* **2021**, *9* (44), 25189–25195. https://doi.org/10.1039/D1TA07436A.

(12) Gan, L.; Chidambaram, A.; Fonquernie, P. G.; Light, M. E.; Choquesillo-Lazarte, D.; Huang, H.; Solano, E.; Fraile, J.; Viñas, C.; Teixidor, F.; Navarro, J. A. R.; Stylianou, K. C.; Planas, J. G. A Highly Water-Stable Meta -Carborane-Based Copper Metal–Organic Framework for Efficient High-Temperature Butanol Separation. *J. Am. Chem. Soc.* **2020**, *142* (18), 8299–

8311. https://doi.org/10.1021/jacs.0c01008.

(13) Mesfer, M. K. Al; Danish, M.; Khan, M. I.; Ali, I. H.; Hasan, M.; Jery, A. El. Continuous Fixed Bed CO2 Adsorption: Breakthrough, Column Efficiency, Mass Transfer Zone. *Processes* **2020**, *8* (10), 1233. https://doi.org/10.3390/pr8101233.

(14) Yang, D.-A.; Cho, H.-Y.; Kim, J.; Yang, S.-T.; Ahn, W.-S. CO 2 Capture and Conversion Using Mg-MOF-74 Prepared by a Sonochemical Method. *Energy Environ. Sci.* **2012**, *5* (4), 6465–6473. https://doi.org/10.1039/C1EE02234B.

(15) Mason, J. A.; Sumida, K.; Herm, Z. R.; Krishna, R.; Long, J. R. Evaluating Metal–Organic Frameworks for Post-Combustion Carbon Dioxide Capture via Temperature Swing Adsorption. *Energy Environ. Sci.* **2011**, *4* (8), 3030. https://doi.org/10.1039/c1ee01720a.

(16) Adhikari, A. K.; Lin, K.-S. Improving CO 2 Adsorption Capacities and CO 2 /N 2 Separation Efficiencies of MOF-74(Ni, Co) by Doping Palladium-Containing Activated Carbon. *Chem. Eng. J.* **2016**, *284*, 1348–1360. https://doi.org/10.1016/j.cej.2015.09.086.

(17) Nuhnen, A.; Janiak, C. A Practical Guide to Calculate the Isosteric Heat/Enthalpy of Adsorption via Adsorption Isotherms in Metal–Organic Frameworks, MOFs. *Dalt. Trans.* **2020**, *49* (30), 10295–10307. https://doi.org/10.1039/D0DT01784A.

(18) Henrotin, A.; Heymans, N.; Duprez, M. E.; Mouchaham, G.; Serre, C.; Wong, D.; Robinson, R.; Mulrooney, D.; Casaban, J.; De Weireld, G. Lab-Scale Pilot for CO2 Capture Vacuum Pressure Swing Adsorption: MIL-160(Al) vs Zeolite 13X. *Carbon Capture Sci. Technol.* **2024**, *12*, 100224. https://doi.org/10.1016/j.ccst.2024.100224.

(19) Nandi, S.; Collins, S.; Chakraborty, D.; Banerjee, D.; Thallapally, P. K.; Woo, T. K.; Vaidhyanathan, R. Ultralow Parasitic Energy for Postcombustion CO 2 Capture Realized in a Nickel Isonicotinate Metal–Organic Framework with Excellent Moisture Stability. *J. Am. Chem. Soc.* **2017**, *139* (5), 1734–1737. https://doi.org/10.1021/jacs.6b10455.

(20) Abánades Lázaro, I.; Mazarakioti, E. C.; Andres-Garcia, E.; Vieira, B. J. C.; Waerenborgh, J. C.; Vitórica-Yrezábal, I. J.; Giménez-Marqués, M.; Mínguez Espallargas, G. Ultramicroporous Iron-Isonicotinate MOFs Combining Size-Exclusion Kinetics and Thermodynamics for Efficient CO 2 /N 2 Gas Separation. *J. Mater. Chem. A* **2023**, *11* (10), 5320–5327. https://doi.org/10.1039/D2TA08934C.

(21) Nguyen, T. T. T.; Lin, J.-B.; Shimizu, G. K. H.; Rajendran, A. Separation of CO2 and N2 on a Hydrophobic Metal Organic Framework CALF-20. *Chem. Eng. J.* **2022**, *442*, 136263. https://doi.org/10.1016/j.cej.2022.136263.

(22) Wen, H.-M.; Liao, C.; Li, L.; Alsalme, A.; Alothman, Z.; Krishna, R.; Wu, H.; Zhou, W.; Hu, J.; Chen, B. A Metal–Organic Framework with Suitable Pore Size and Dual Functionalities for Highly Efficient Post-Combustion CO 2 Capture. *J. Mater. Chem. A* **2019**, *7* (7), 3128–3134. https://doi.org/10.1039/C8TA11596F.

(23) Chen, B.; Fan, D.; Pinto, R. V.; Dovgaliuk, I.; Nandi, S.; Chakraborty, D.; García-Moncada, N.; Vimont, A.; McMonagle, C. J.; Bordonhos, M.; Al Mohtar, A.; Cornu, I.; Florian, P.; Heymans, N.; Daturi, M.; De Weireld, G.; Pinto, M.; Nouar, F.; Maurin, G.; Mouchaham, G.; Serre, C. A Scalable Robust Microporous Al-MOF for Post-Combustion Carbon Capture. *Adv. Sci.* **2024**, *11* (21). https://doi.org/10.1002/advs.202401070.

(24) Liu, S.; Yao, S.; Liu, B.; Sun, X.; Yuan, Y.; Li, G.; Zhang, L.; Liu, Y. Two Ultramicroporous Metal–Organic Frameworks Assembled from Binuclear Secondary Building Units for Highly Selective CO 2 /N 2 Separation. *Dalt. Trans.* **2019**, *48* (5), 1680–1685.

https://doi.org/10.1039/C8DT04424D.

(25) Gan, L.; Andres-Garcia, E.; Mínguez Espallargas, G.; Planas, J. G. Adsorptive Separation of CO 2 by a Hydrophobic Carborane-Based Metal–Organic Framework under Humid Conditions. *ACS Appl. Mater. Interfaces* **2023**, *15* (4), 5309–5316. https://doi.org/10.1021/acsami.2c20373.

(26) Vismara, R.; Terruzzi, S.; Maspero, A.; Grell, T.; Bossola, F.; Sironi, A.; Galli, S.; Navarro, J. A. R.; Colombo, V. CO 2 Adsorption in a Robust Iron(III) Pyrazolate-Based MOF: Molecular-Level Details and Frameworks Dynamics From Powder X-ray Diffraction Adsorption Isotherms. *Adv. Mater.* **2024**, *36* (12). https://doi.org/10.1002/adma.202209907.

(27) Herm, Z. R.; Wiers, B. M.; Mason, J. A.; van Baten, J. M.; Hudson, M. R.; Zajdel, P.; Brown, C. M.; Masciocchi, N.; Krishna, R.; Long, J. R. Separation of Hexane Isomers in a Metal-Organic Framework with Triangular Channels. *Science (80-.).* **2013**, *340* (6135), 960–964. https://doi.org/10.1126/science.1234071.

(28) Shekhah, O.; Belmabkhout, Y.; Chen, Z.; Guillerm, V.; Cairns, A.; Adil, K.; Eddaoudi, M. Made-to-Order Metal-Organic Frameworks for Trace Carbon Dioxide Removal and Air Capture. *Nat. Commun.* **2014**, *5* (1), 4228. https://doi.org/10.1038/ncomms5228.

(29) Shi, Y.; Xie, Y.; Cui, H.; Alothman, Z. A.; Alduhaish, O.; Lin, R.-B.; Chen, B. An Ultramicroporous Metal–Organic Framework with Dual Functionalities for High Sieving Separation of CO2 from CH4 and N2. *Chem. Eng. J.* **2022**, *446*, 137101. https://doi.org/10.1016/j.cej.2022.137101.

(30) Li, Z.; Shi, K.; Zhai, L.; Wang, Z.; Wang, H.; Zhao, Y.; Wang, J. Constructing Multiple Sites of Metal-Organic Frameworks for Efficient Adsorption and Selective Separation of CO2. *Sep. Purif. Technol.* **2023**, *307*, 122725. https://doi.org/10.1016/j.seppur.2022.122725.

(31) Sagastuy-Breña, M.; Mileo, P. G. M.; Sánchez-González, E.; Reynolds, J. E.; Jurado-Vázquez, T.; Balmaseda, J.; González-Zamora, E.; Devautour-Vinot, S.; Humphrey, S. M.; Maurin, G.; Ibarra, I. A. Humidity-Induced CO 2 Capture Enhancement in Mg-CUK-1. *Dalt. Trans.* **2018**, *47* (44), 15827–15834. https://doi.org/10.1039/C8DT03365J.

(32) Chen, X.; Leng, J.; Ma, F.; Wu, J.; Jin, Y.; Yu, M.; Huang, H.; Shang, S.; Ye, D. Highly Controllable CO2 Capture Performance under Varied Humidity Conditions by Finely Tuned Metal and Organic Ligand Compositions of DMOF Adsorbents. *Microporous Mesoporous Mater.* **2025**, *389*, 113559. https://doi.org/10.1016/j.micromeso.2025.113559.

(33) Capelo-Avilés, S.; de Fez-Febré, M.; Balestra, S. R. G.; Cabezas-Giménez, J.; Tomazini de Oliveira, R.; Gallo Stampino, I. I.; Vidal-Ferran, A.; González-Cobos, J.; Lillo, V.; Fabelo, O.; Escudero-Adán, E. C.; Falvello, L. R.; Parra, J. B.; Rumori, P.; Turnes Palomino, G.; Palomino Cabello, C.; Giancola, S.; Calero, S.; Galán-Mascarós, J. R. Selective Adsorption of CO2 in TAMOF-1 for the Separation of CO2/CH4 Gas Mixtures. *Nat. Commun.* **2025**, *16* (1), 3243. https://doi.org/10.1038/s41467-025-58426-w.

(34) Qazvini, O. T.; Babarao, R.; Telfer, S. G. Selective Capture of Carbon Dioxide from Hydrocarbons Using a Metal-Organic Framework. *Nat. Commun.* **2021**, *12* (1), 197. https://doi.org/10.1038/s41467-020-20489-2.

(35) Wu, X.; Yuan, B.; Bao, Z.; Deng, S. Adsorption of Carbon Dioxide, Methane and Nitrogen on an Ultramicroporous Copper Metal–Organic Framework. *J. Colloid Interface Sci.* **2014**, *430*, 78–84. https://doi.org/10.1016/j.jcis.2014.05.021.

(36) Chen, K.; Madden, D. G.; Pham, T.; Forrest, K. A.; Kumar, A.; Yang, Q.; Xue, W.; Space, B.;

Perry, J. J.; Zhang, J.; Chen, X.; Zaworotko, M. J. Tuning Pore Size in Square-Lattice Coordination Networks for Size-Selective Sieving of CO 2. *Angew. Chemie Int. Ed.* **2016**, *55* (35), 10268–10272. https://doi.org/10.1002/anie.201603934.

(37) Oggianu, M.; Manna, F.; Mameli, V.; Cannas, C.; Guiotto, V.; Crocellà, V.; Quesada, S.; Sassone, D.; Sacco, A.; Gallo Stampino, I. I.; de Oliveira, R. T.; Capelo, S.; Galan-Mascaros, J. R.; Masciocchi, N.; Mercuri, M. L. A 3D Polymorphic Cu-Based Ultramicroporous MOF Capable of CO 2 Uptake and Conversion. *J. Mater. Chem. A* **2026**. https://doi.org/10.1039/D5TA09255H.

(38) Hazen, R.; Finger, L. *ComparativeCrystalChemistry*; Wiley, Ed.; Chichester (UK), 1982.
